# Acorn‐Weevil Interactions in Semi‐Humid Evergreen Broad‐Leaved Forests in Yunnan, China: Trade‐Offs Among Acorn Functional Traits

**DOI:** 10.1002/ece3.72045

**Published:** 2025-08-21

**Authors:** Shengquan Fang, Chongyun Wang, Shaoji Hu, Mingchun Peng, Yongping Li, Chunyan Lan, Xinrong Li, Dengpeng Chen, Biao Zhao

**Affiliations:** ^1^ State Key Laboratory of Vegetation Structure, Function and Construction (VegLab) Yunnan University Kunming Yunnan People's Republic of China; ^2^ Institute of Ecology and Geobotany, School of Ecology and Environmental Science Yunnan University Kunming Yunnan People's Republic of China; ^3^ Yuxi Institute of Ecology and Environment Science Yuxi Yunnan People's Republic of China; ^4^ Institute of International Rivers and Eco‐Security Yunnan University Kunming Yunnan People's Republic of China; ^5^ Yunnan Key Laboratory of International Rivers and Transboundary Eco‐security Yunnan University Kunming People's Republic of China; ^6^ School of Agriculture Yunnan University Kunming Yunnan People's Republic of China

**Keywords:** acorn weevils, predation, semi‐humid evergreen broad‐leaved forests, trade‐offs acorn functional traits

## Abstract

Acorns are crucial for the regeneration and stability of oak forest communities, yet they often suffer significant predation by weevils before dispersal. Understanding the interaction between acorn functional traits and weevil infestation is essential for elucidating plant‐insect coevolution. This study examined the relationship between acorn functional traits and weevil infestation rates in six dominant Fagaceae species (*Quercus schottkyana*, *Q. delavayi*, *Q. franchetii*, *Castanopsis delavayi*, *C. orthacantha* and *Lithocarpus dealbatus*) in semi‐humid evergreen broad‐leaved forests in Yunnan, China. The results showed significant interspecific variation in acorn traits and infestation rates. *L. dealbatus* had the lowest infestation rate (9%), associated with its thick pericarp (0.88 mm), while *Quercus* species with higher levels of secondary metabolites (total phenols, flavonoids and tannins) exhibited higher infestation rates (34%–51%). *Castanopsis* species maintained moderate infestation rates with a combination of moderate physical defences and high starch content. Mixed‐effects models revealed that morphological and chemical traits together explained 38% of the variation in infestation rates, with pericarp thickness and tannin content being key inhibitory factors. Infested acorns upregulated secondary metabolite concentrations as a defensive response, reflecting phenotypic plasticity. Standardised major axis regression confirmed stable trade‐offs between physical and chemical defences, as well as between defence compounds and nutrient reserves. Even in the absence of weevil infestation, resource allocation strategies were inherent. We propose a three‐tier defence model: physical barriers (pericarp/cicatrix), chemical defences (total phenols/tannins) and nutrient regulation (starch reallocation). These complementary strategies collectively maintain forest community stability. The study provides new insights into plant–animal coevolution and supports conservation efforts for semi‐humid evergreen broad‐leaved forests.

## Graphical abstract

This study investigates the relationship between acorn functional traits and weevil infestation rates in six dominant Fagaceae species in semi‐humid evergreen broad‐leaved forests in Yunnan, China. Morphological traits (pericarp thickness) and chemical defence compounds (tannin content) are key factors influencing weevil infestation rates. Infested acorns respond by increasing secondary metabolite concentrations. The study proposes a three‐tier defence model—physical barriers, chemical defences and nutrient regulation—which collectively maintain forest community stability.

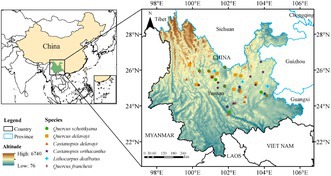

## 1 Introduction

Plant functional traits influence individual fitness in variable environments by regulating growth, survival and reproductive yield (Reich 1995; Cornelissen et al. 2003; Violle et al. 2007). Seeds are intergenerational carriers of genetic variation in seed plants and the study of seed functional traits is crucial for a better understanding of plant population biology, resource conservation, plant–animal interactions and ecosystem function (Amartuvshin et al. 2019). Jiménez‐Alfaro et al. (2016) compiled a nonexhaustive list of seed functional traits and classified them into two practical categories: morphological and chemical traits (Jiménez‐Alfaro et al. 2016). These measurable traits provide ecological features of the functional characteristics of plant communities. They help researchers study plant–animal interactions and show that studying functional traits is becoming essential for understanding ecological patterns and evolutionary processes in plant communities (Fricke and Wright 2016; Amartuvshin et al. 2019).

Acorns are hard‐shelled fruits produced by various oak species, and the seeds inside are crucial for the regeneration of oak forests (Vinha et al. 2020; Inácio et al. 2024). Multiple weevil species (Coleoptera: Curculionoidea), particularly those of the genus *Curculio*, are primary consumers of acorns before dispersal, impacting seed availability for oak forest regeneration (Keeley 1991; Siscart et al. 1999; Muñoz et al. 2014; Wang et al. 2014; Xia et al. 2016; Chen et al. 2020; Villalobos et al. 2023). Most of the consumed acorns are unable to germinate due to depletion from foraging, which significantly reduces seed supply. Additionally, weevil feeding enables the entry of other organisms such as fungi and bacteria into the acorns, further reducing their chances of survival (Williams and Hawkins 2020). This reduction affects species coexistence within a community and determines the spatial–temporal patterns of the community in the future (Pakeman 2001). When seed amount drops below a certain threshold, it can threaten the regeneration of plant populations (Castro et al. 1999). Acorn functional traits (AFTs) are strongly related to the success of colonisation and significantly influence the diversity, survival, interspecific interactions and population regeneration of Fagaceae species (Willis et al. 2014; Malav et al. 2015; Méndez‐Tovar et al. 2015). AFTs vary among oak species and influence weevil foraging preferences (Yi and Yang 2010; Muñoz et al. 2014; Bartlow et al. 2018). Variation in AFTs is influenced by abiotic factors (W. Q. Sun 1999; Jansen et al. 2006; Deng et al. 2018), while at the same time, it is driven by biotic factors such as acorn size preferences, oviposition timing and weevil foraging behaviour (Daws et al. 2005; Braga et al. 2018). Many studies have shown that AFTs play an important role in the ecological characteristics and processes of oak forests (McGill et al. 2006; Dey 2014; Laughlin 2014a, 2014b; Pierce et al. 2014; Sun 2021).

Contrasting selective pressures can act on interactions between weevils and oaks within complex evolutionary scenarios on both sides (Crawley and Long 1995; Bonal et al. 2007; Muñoz et al. 2014). Plants have differences in seed abundance (plant satiation) or in the ability of seeds to survive insect infestation (seed satiation) (the predator satiation hypothesis, e.g., Yi and Yang 2010; Espelta, Bonal, and Sánchez‐Humanes 2009; Espelta, Cortés, et al. 2009; Espelta et al. 2017). Coexistence of multiple weevil species is constrained by available acorns and leads to morphological differences among weevil species, such as their adaptation to the oviposition site (Hughes and Vogler 2004). AFTs help us understand the multi‐dimensional spatial functions of plant traits, co‐evolution relationships with animal foraging and the renewal of plant communities (McGill et al. 2006). There are more studies on trade‐offs between acorn defensive traits and impacts on interaction patterns between acorns and rodents in forest ecosystems (Xiao et al. 2003; Chen et al. 2012; Zhang et al. 2016, 2024; Zaret et al. 2024). There is still less focus on the trade‐off between morphological and chemical defensive traits of acorn associated with pre‐dispersal predation by weevils, as well as the allocation strategy between nutrients and defence substances (Yang et al. 2023).

Semi‐humid evergreen broad‐leaved forests (SEBFs) are a unique and endemic species‐rich vegetation type in southwest China (Wu and Zhu 1987). As the horizontal zonal vegetation type on the Yunnan Plateau, it represents the primary forest ecosystem and plays a crucial role in the maintenance of biodiversity and ecological resilience. The dominant or co‐dominant species in the arboreal layer of SEBFs are mainly six oak species (s.l.), that is, *Quercus schottkyana*, *Q. delavayi*, *Q. franchetii*, *Castanopsis delavayi*, *C. orthacantha* and *Lithocarpus dealbatus*, which are sympatrically distributed in this region (Wu and Zhu 1987; Tang 2015; Zhu and Tan 2022). Due to historical deforestation and long‐term exploitation (such as logging and rural development), most of the preserved forests are isolated patches or fragmented areas in the form of sprouting scrubs (Li et al. 2012; Zhu and Tan 2022; Zuo et al. 2023). Old‐growth SEBFs with large areas can only be found near temples, village sacred hills, or water resources due to conservation purposes (Wu and Zhu 1987). Even in these mature forests, the oak tree often lacks seedlings and young trees, exhibiting poor self‐regeneration (Zhu and Tan 2022). Research shows that weevil foraging on acorns is a major factor hindering the regeneration of oak forests, especially during the acorn falling season (Méndez‐Tovar et al. 2015; Xia et al. 2016). For *Quercus schottkyana* and *Castanopsis delavayi*, the weevil infestation rate is approximately 25% (Wang et al. 2014); in similar holm oak forests of the Mediterranean, this rate can reach 100% for individual species (Siscart et al. 1999). Pre‐dispersal predation may contribute to differences in seed supply and ultimately in seedling recruitment between co‐existing oaks (Espelta, Bonal, and Sánchez‐Humanes 2009; Espelta, Cortés, et al. 2009). For acorns, due to the wide distribution of SEBFs and significant differences in hydrothermal conditions, AFTs vary obviously (Bakis and Babaç 2015). For example, the morphological traits of acorns in *Quercus* (section *Cyclobalanopsis*) exhibit an obvious tripartite zonal distribution pattern (W. Q. Sun 1999; Jansen et al. 2006; Deng et al. 2018); acorns of *Castanopsis* are typically enclosed in spiny or thick cupules (Wu 1998). The variation in these AFTs also influences the feeding choices of weevils (Crawley and Long 1995; Muñoz et al. 2014). However, to date, although some studies have been carried out on the species of acorn weevils in SEBFs, the feeding of weevils and its relationship with AFTs remain unclear (Wang et al. 2014; Fang et al. 2025). This will be an obstacle in forest management for the persistence and health of SEBFs.

In this study, we hypothesised that co‐fruiting oak trees with different AFTs in SEBFs would exhibit varying degrees of susceptibility to pre‐dispersal seed predation by weevils. Given the potential for multiple weevil species with different host preferences, we aimed to elucidate the specific resistance mechanisms in AFTs that influence weevil infestation rates. Specifically, we explored the following questions: (1) What is the status of AFTs and weevil infestation rates in dominant oak species within SEBFs? (2) What is the relationship between weevil infestation rates and AFTs? (3) How do AFTs trade off against weevil feeding?

## 2 Materials and Methods

### 2.1 Study Area and Field Sampling

SEBFs are mainly distributed 1500–2500 m of the Central Yunnan Plateau in southwestern China, and they are mostly fragmented (Wu and Zhu 1987). These forests are typically dominated by single‐species oak stands, with only one dominant oak species present in most areas. However, in some regions, multiple oak species coexist as dominant species. SEBFs are situated in the Southwest monsoon region, which experiences a dry season from October to May in the next year (Jin 1979; Song and Da 2016). Additionally, this region is characteristic of a mountainous climate, with an average annual temperature of 13°C–16°C and average annual precipitation of 800–1000 mm (Yang 1990; Meng et al. 2001; Xiao et al. 2019).

**FIGURE 1:**
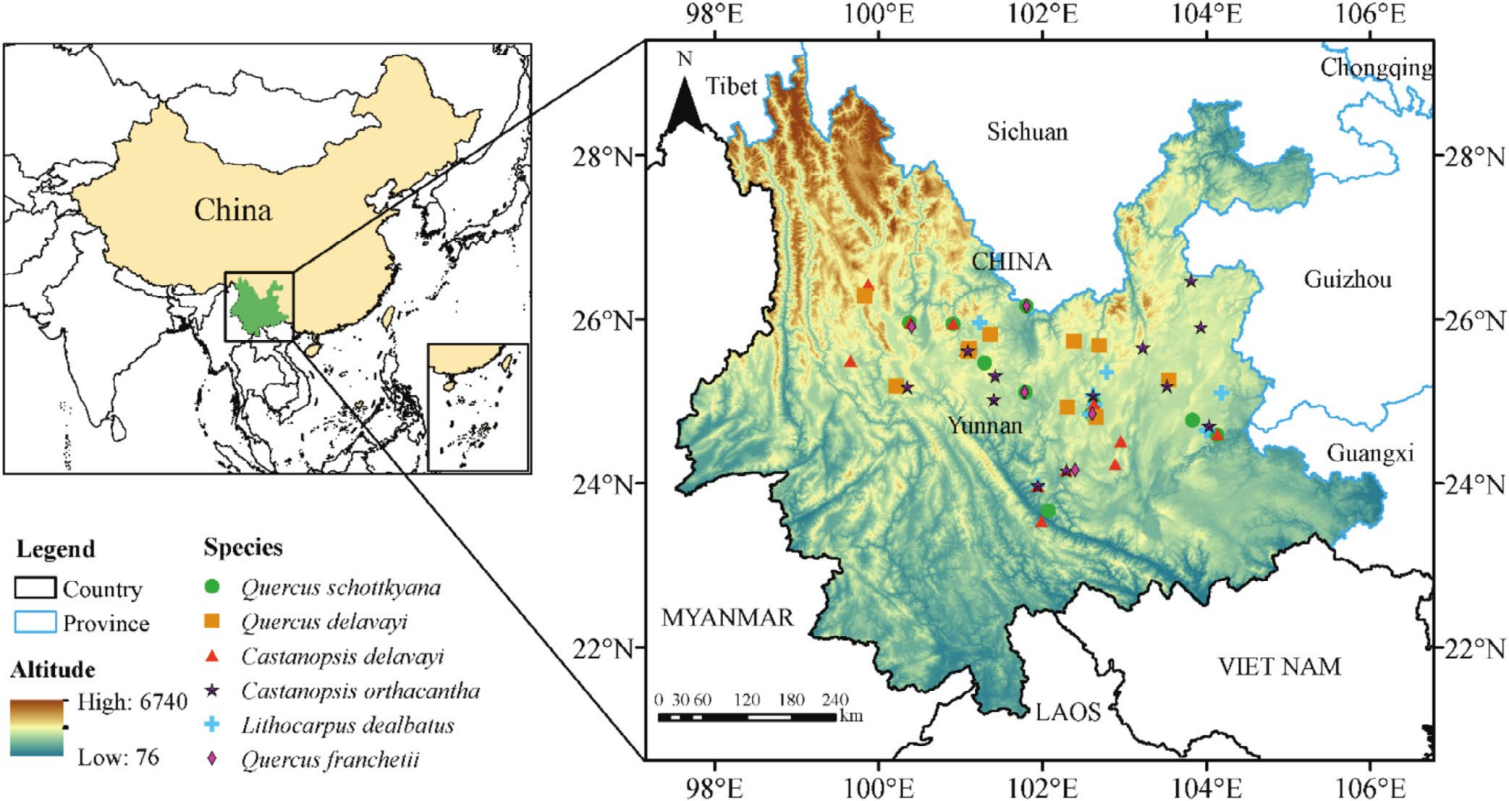
Distribution of sampling sites for acorns of six dominant Fagaceae species in SEBFs.

In 2022 and 2023, during the acorn falling season from October to November, we sampled 17,885 acorns from 60 dominant oak tree populations in SEBFs, which encompassed six dominant oak species (Figure 1, Table S1). In each forest population, we randomly selected 3–5 mother trees, ensuring that the distance between sampled trees was greater than 10 m. Mature acorns were collected beneath the crown, including those that had fallen on the ground and had not yet detached from the branches. During the sampling process, we carefully inspected each acorn to ensure that only those without obvious physical damage were collected. They were labelled by location and species. The sampled acorns were then transported to the laboratory and stored in a refrigerator at 4°C for subsequent measurements and assays.

### 2.2 Investigation of Acorn Infection

To analyse the degree of intactness and weevil infection in acorns, we selected 50 mature acorns from each species population using the quadrat method for assessment (Tattoni et al. 2021). Because some populations contained fewer than 50 acorns, we included all available acorns in the measurements. A total of 2578 acorns from 52 populations were used for testing. Each selected acorn was initially examined for the integrity of its outer pericarp and for any identified insect holes. The presence of holes indicated weevil infestation. If there were no surface insect holes in the outer pericarp, we used a scalpel to cut open the outer pericarp and observed the seeds inside. If the seeds had no insect holes outside and were intact inside, they were classified as intact; if eggs or larvae were present or if the seeds had been consumed, they were classified as infested (Figure S1) (Espelta, Bonal, and Sánchez‐Humanes 2009; Espelta, Cortés, et al. 2009; Kellner et al. 2014; Canelo et al. 2021). The number of acorns in different states was recorded and quantified using the formula, which illustrates the insect infestation rate: $R_{\text{infested}}=N_{\text{infested}}/N_{\text{acorn}}\times 100\%$, where *R*_infested_ represents infestation rate, *N*_infested_ represents infested acorns and *N*_acorn_ for the total number of acorns measured (Canelo et al. 2021). During the count, we used a Canon 80D camera to document the morphology of both intact and weevil‐infested acorns (Figure S2).

### 2.3 Measurements of Acorn Functional Traits

#### 2.3.1 Acorn Morphological Traits

All acorns assessed as intact and weevil‐infested (treatments) in Section 2.2 were measured for five morphological traits, including acorn mass, volume, fruit shape index, pericarp thickness and cicatrix (defined as the scar tissue where the acorn attaches to the cupule and branch, Figure S3) thickness (Liu 2016; Woziwoda et al. 2023). During field sampling, insect holes were observed on the cicatrix of acorns, indicating that weevils may enter or exit through this area (Figure S2—LD2). This finding led us to include cicatrix thickness and pericarp thickness as key physical defence traits to evaluate their role in resisting weevil penetration. We used an analytical balance (BSA223S, OLABO, Shandong, China) to measure the acorn mass with an accuracy of 0.001 g. The transverse and longitudinal diameters of the acorns were measured using digital vernier callipers (DELL, Zhejiang, China) with an accuracy of 0.01 mm, and fruit shape index was calculated using the formula: FSI=LD/TD, where FSI represents fruit shape index, LD represents longitudinal diameter of the acorn, and TD represents the transverse diameter of the acorn. Acorn volumes were determined using the drainage method and alongside data from acorn transverse and longitudinal diameters (Table S2). After cutting the pericarp of both intact and weevil‐infested acorns with a scalpel, we measured the physical defence traits (pericarp, cicatrix) using a thickness gauge (SYNTEK, Zhejiang, China), with the middle of the pericarp or cicatrix serving as the standard site (Figure S3), and with an accuracy of 0.01 mm.

#### 2.3.2 Acorn Chemical Traits

Experimental material was taken from the acorns after morphological trait measurements were completed in Section 2.3.1. However, as the number of weevil‐infested acorns in some populations was fewer than five, and to standardise the measurement scale, it was ensured that at least five intact acorns and five weevil‐infested acorns (treatments) were measured for chemical traits in each population. We measured the chemical traits of all eligible acorns, focusing on several key classes of compounds. They included water content, starch content as a nutrient, and the content of three secondary metabolites (chemical defence substances), namely total phenols, total flavonoids and tannins (Shimada and Saitoh 2006). A total of 2400 samples (2 treatments × 48 populations × 5 classes of compounds × 5 replicates = 2400 samples) were measured for chemical traits. Before measurement, we dried the sample acorns by baking them in an oven at 70°C for 4.5 h, followed by drying at (103°C ± 1°C) for 17–18 h until they reached a constant weight, after that they were removed. We measured the water content of the acorns using the formula: $\mathrm{WC}=\frac{{\mathrm{AC}}_{F}-{\mathrm{AC}}_{D}}{{\mathrm{AC}}_{F}}\times 100\%$, where WC represents the water content of acorns, AC_F_ is the fresh weight of acorns and AC_D_ is the dry weight of acorns (Sun 2021). Next, we ground the samples into powder using a benchtop homogeniser (FastPrep‐24, MP Biomedicals, America) and a mortar and pestle. After sieving through a 40–60 mesh, the samples were stored at −80°C in a freezer for subsequent assays. The chemical kits used for measuring the content of compounds were purchased from Suzhou Grace Biotechnology Co. Ltd., and the extraction of each chemical substance was carried out according to the methods outlined in the chemical kits, with final examinations conducted using enzyme linked immunosorbent assay (ELISA).

### 2.4 Data Analysis

We performed all statistical analyses using R version 4.3.1 (R Core Team 2022). Firstly, we compared acorn status, intact acorn's functional traits, and functional traits between intact and infested acorns among six dominant oak species of SEBFs. We used the Shapiro–Wilk test and Levene's test (package *car* (Fox and Weisberg 2019)) to assess the distribution and homogeneity of the data, respectively, and found that none of the data regarding acorn status and functional traits were normally distributed or homogeneous. Therefore, we used the Kruskal–Wallis test and Dunn's multiple comparison test (package *FSA* (Ogle et al. 2023)) to compare the acorn status and functional traits among different species, correcting for significance using the Benjamini‐Hochberg method. Additionally, we used the PCA function from the package *FactoMineR* (Husson et al. 2017) to conduct principal component analyses on selected AFTs variables. This approach aimed to avoid clustering of a priori known strong correlations, reduce the dimensionality of the dataset, and thereby enhance the interpretability of the data (Jolliffe and Cadima 2016). We used Spearman correlations to examine the relationships among AFTs (both morphological and chemical) and their associations with acorn infestation rates. The trait data were log‐transformed (log10) before testing. We fitted a mixed‐effects model using the lmer function from the package *lme4* (Bates et al. 2015) to predict the functional traits affecting infestation rates. In this analysis, we used acorn infestation rates from different populations as the response variable, each AFT (morphological and chemical) as a fixed effect, and the species of acorn, test individual, and sampling site as random effects for prediction. We then used package *glmm.hp* (Lai et al. 2023) to decompose the degree of contribution of morphological and chemical traits within the mixed model. To test whether there is a trade‐off between mechanical/physical and chemical defence development in acorns, we used standardised major axis (SMA) regression (package *smart3*) (Warton et al. 2012) for this exploration.

## 3 Results

### 3.1 Infestation Rates, AFTs and Relationships at Interspecific Level

#### 3.1.1 Interspecific Variation in Infestation Rates

**FIGURE 2:**
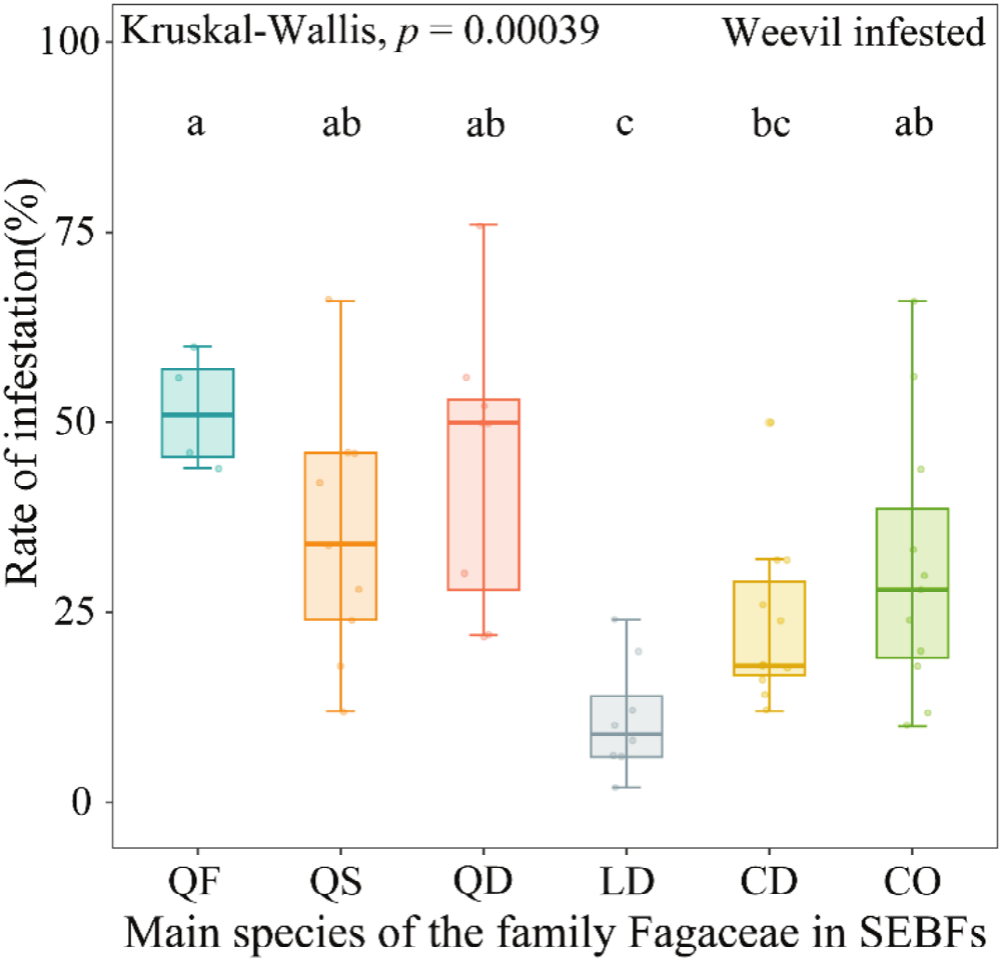
Differences in acorn infestation rates among the six dominant oak species in SEBFs. CD, *Castanopsis delavayi*; CO, *Castanopsis orthacantha*; LD, *Lithocarpus dealbatus*; QD, *Quercus delavayi*; QF, *Quercus franchetii*; QS, *Quercus schottkyana*. The same applies below. Lowercase letters are differences in infestation rates between acorns of different species, and the same letter means no significant difference.

Significant interspecific differences in acorn infestation rates were observed among the six dominant oak species (*H* = 22.661, df = 5, *p* < 0.001). Specifically, *Lithocarpus dealbatus* exhibited the lowest infestation rate (9%), which was significantly lower than the other five species. Within the *Quercus* genus, *Q. franchetii* showed the highest infestation rate (51%), followed by *Q. delavayi* (50%) and *Q. schottkyana* (34%). Notably, no significant difference in infestation rates was found between the two *Castanopsis* species (*C. delavayi*: 18%; *C. orthacantha*: 28%) (Figure 2, Table S3).

#### 3.1.2 Differences and Association of ATFs

**FIGURE 3:**
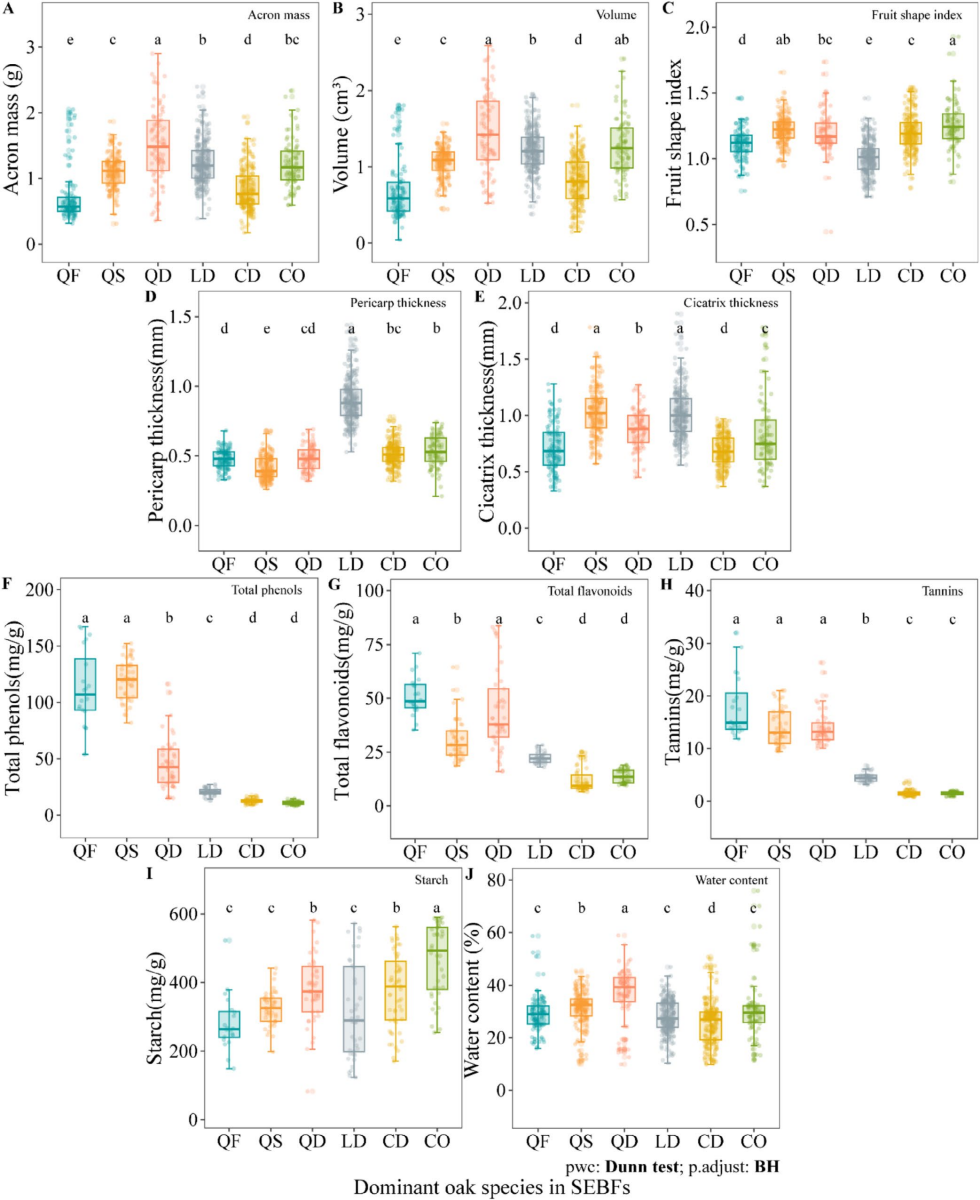
Interspecific differences in acorn functional traits of the six dominant oak species in SEBFs. Species abbreviations and lowercase letters have the same meanings as in Figure 2. (A) Acorn mass; (B) Acorn volume; (C) Fruit shape index; (D) Pericarp thickness; (E) Cicatrix thickness; (F) Total phenols; (G) Total flavonoids; (H) Tannins; (I) Strach; (J) Water content.

The six species exhibited significant interspecific variation in AFTs (Figure 3, Table S4). Morphologically, *Q. delavayi* possessed the largest acorn volume (1.42 cm^3^), being 2.4‐fold greater than that of *Q. franchetii* (0.59 cm^3^). Regarding defensive traits, *L. dealbatus* showed significantly thicker pericarps (0.88 mm) than other species, measuring 2.3 times thicker than those of *Q. schottkyana* (0.39 mm). Chemical defences demonstrated even more pronounced interspecific differences, with *Q. franchetii* containing 10.7 times higher total phenols (112.64 mg/g) than *C. orthacantha* (10.54 mg/g).

**FIGURE 4:**
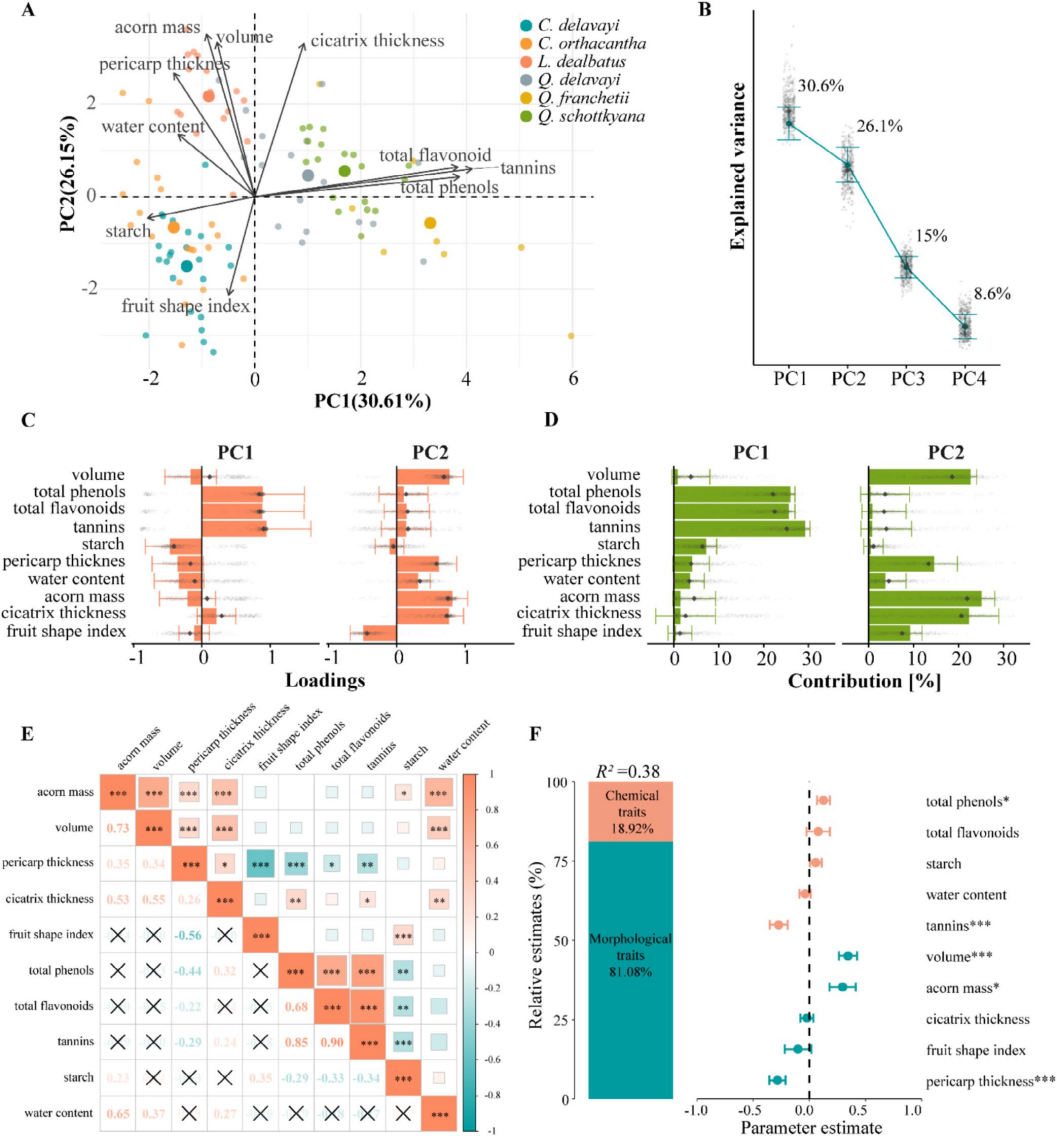
Principal component analysis (A–D), spearman correlations of AFTs (E) and mixed‐effects models based on AFTs and infestation rates (F). (A) Two‐label plot obtained from PCA, with different coloured points representing different oak species of SEBFs. (B) Differences in retained principal components. (C) Loadings bar plot and (D) variable contribution to retained principal components. In B to D, solid circles and bars represent estimates from complete data; error bars centred on estimates indicate standard errors from bootstrap procedures with 499 iterations; small grey diamonds represent estimates for each bootstrap iteration, and large grey diamonds represent medians across all bootstrap iterations. (E) Colour and numbers reflect the magnitude and direction of correlation coefficients. ‘×’ marks non—significant groups. (F) Left side shows percentage of explained variance for morphological and chemical traits; right side shows mean parameter estimates (standardised regression coefficients) with 95% confidence intervals. *p < 0.05; **p < 0.01; ***p < 0.001.

Principal component analysis (PCA) further revealed interspecific multidimensional differentiation in AFTs (Figure 4A–D, Table S5): the first three principal components accounted for 71.79% of the variation. PC1 (30.61%) was driven by chemical defence traits such as tannins (loading = 0.947) and total phenols (0.892), while PC2 (26.15%) was driven by morphological traits including acorn mass (0.811) and pericarp thickness (0.617). There was a significant separation in species distribution: *Quercus* species clustered in the high‐value region of PC1 (strong chemical defence), *Lithocarpus* species had the highest scores on PC2 (strong physical defence), and *Castanopsis* species was positioned in the intermediate region.

Correlation analysis of AFTs showed that among morphological traits, acorn volume was strongly positively correlated with mass (*R* = 0.73, *p* < 0.001). Among chemical traits, total phenols, flavonoids and tannins were all strongly positively correlated with each other (*p* < 0.001); starch was negatively correlated with these three secondary metabolites (*R* = −0.29 to −0.34, *p* < 0.01). Between morphological and chemical traits, pericarp thickness was also negatively correlated with secondary metabolites (*R* = −0.22 to −0.44, *p* < 0.05) (Figure 4E).

#### 3.1.3 Association Between AFTs and Infestation Rates

These trait variations corresponded with infestation rates across species—*L. dealbatus* with the thickest pericarps exhibited the lowest infestation rate (9%), while *Q. franchetii* with the highest chemical defence concentrations showed the highest infestation (51%) (Figure 2). Mixed‐effects modelling revealed further that morphological and chemical traits collectively explained 38% of the variation in infestation rates, with morphological and chemical traits accounting for 81.08% and 18.92% (Figure 4F, Table S6). Among morphological traits, both acorn volume (*p* < 0.001) and mass (*p* = 0.013) showed significant positive associations with infestation rates, indicating preferential selection by weevils for larger acorns. In contrast, pericarp thickness demonstrated the strongest negative effect (*p* < 0.001), while cicatrix thickness (*p* = 0.706) and fruit shape index (*p* = 0.404) showed no significant effects. For chemical traits, total phenols exhibited marginally significant positive correlation (*p* = 0.029), whereas tannin content showed significant negative correlation (*p* = 0.001). This suggests that a higher total phenol content in the acorns, or a lower tannin content, is associated with a higher rate of weevil infestation. This contrasting pattern may reflect differential defensive functions among phenols. Neither starch content (*p* = 0.286) nor water content (*p* = 0.456) significantly predicted infestation rates.

### 3.2 Infestation Rates, AFTs and Relationships at Intraspecific Level

#### 3.2.1 Association Between Intraspecific Variation and Infestation Rates

**FIGURE 5:**
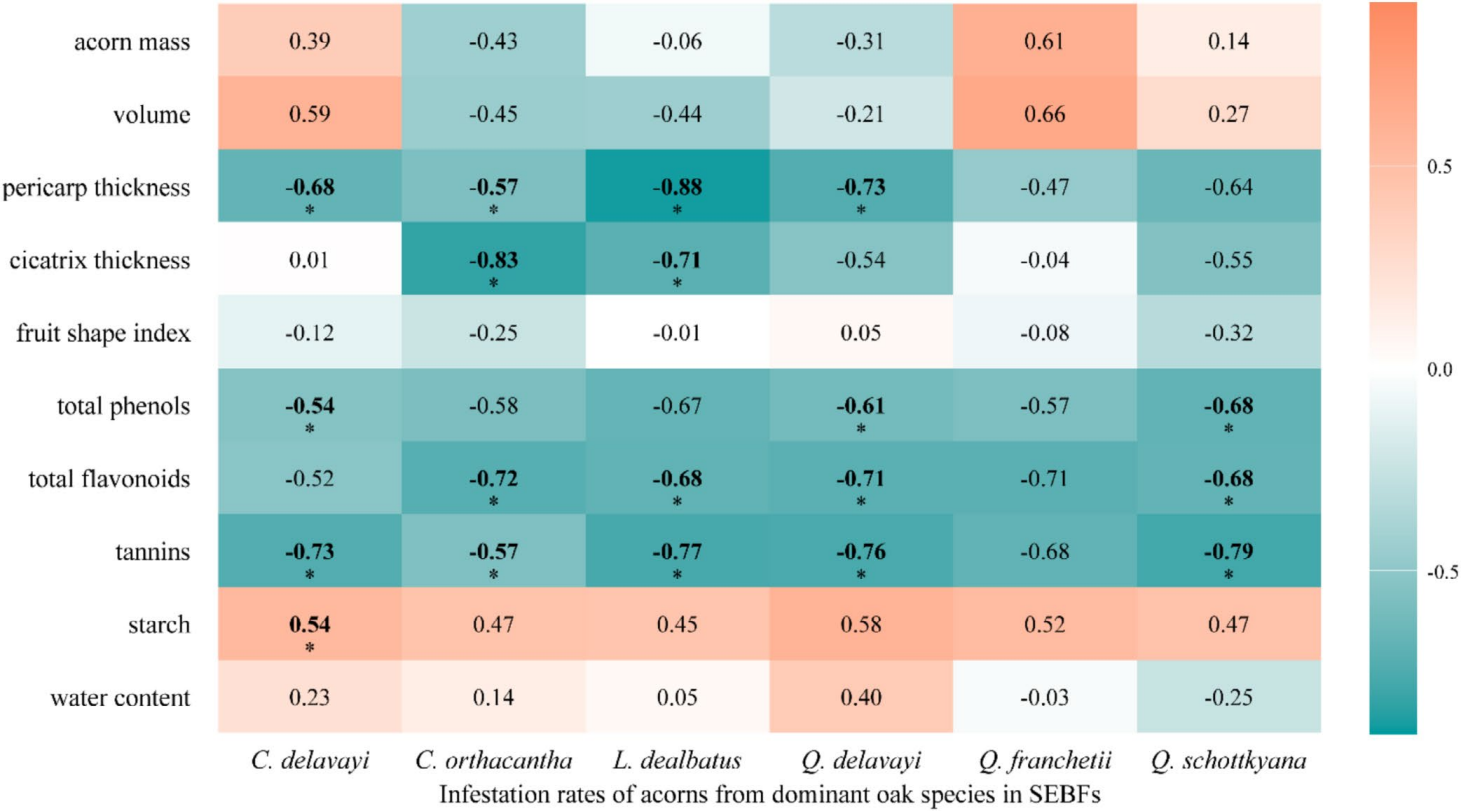
Spearman correlation between acorn functional traits and infestation rates of the six dominant oak species in SEBFs. The numbers represent the correlation coefficients. *p < 0.05.

Spearman's correlation analysis demonstrated species‐specific effects of intraspecific trait variation on infestation rates (Figure 5, Table S7). For physical defences, pericarp thickness showed significant negative correlations with infestation rates in *C. orthacantha* (*R* = −0.57, *p* = 0.02) and *L. dealbatus* (*R* = −0.88, *p* = 0.02), but not in other species. Regarding chemical defences, *Q. delavayi* and *Q. schottkyana* exhibited the most pronounced negative correlations, with all three measured chemical defence compounds showing significant negative relationships with infestation rates. Particularly noteworthy was the significant positive correlation between starch content and infestation rate in *C. delavayi* (*R* = 0.54, *p* = 0.02), consistent with predictions from optimal foraging theory. These results demonstrate that even at intraspecific levels, continuous variation in functional traits can significantly influence weevil host selection behaviour.

#### 3.2.2 Differences in AFTs Between Intact and Infested Acorns

**FIGURE 6:**
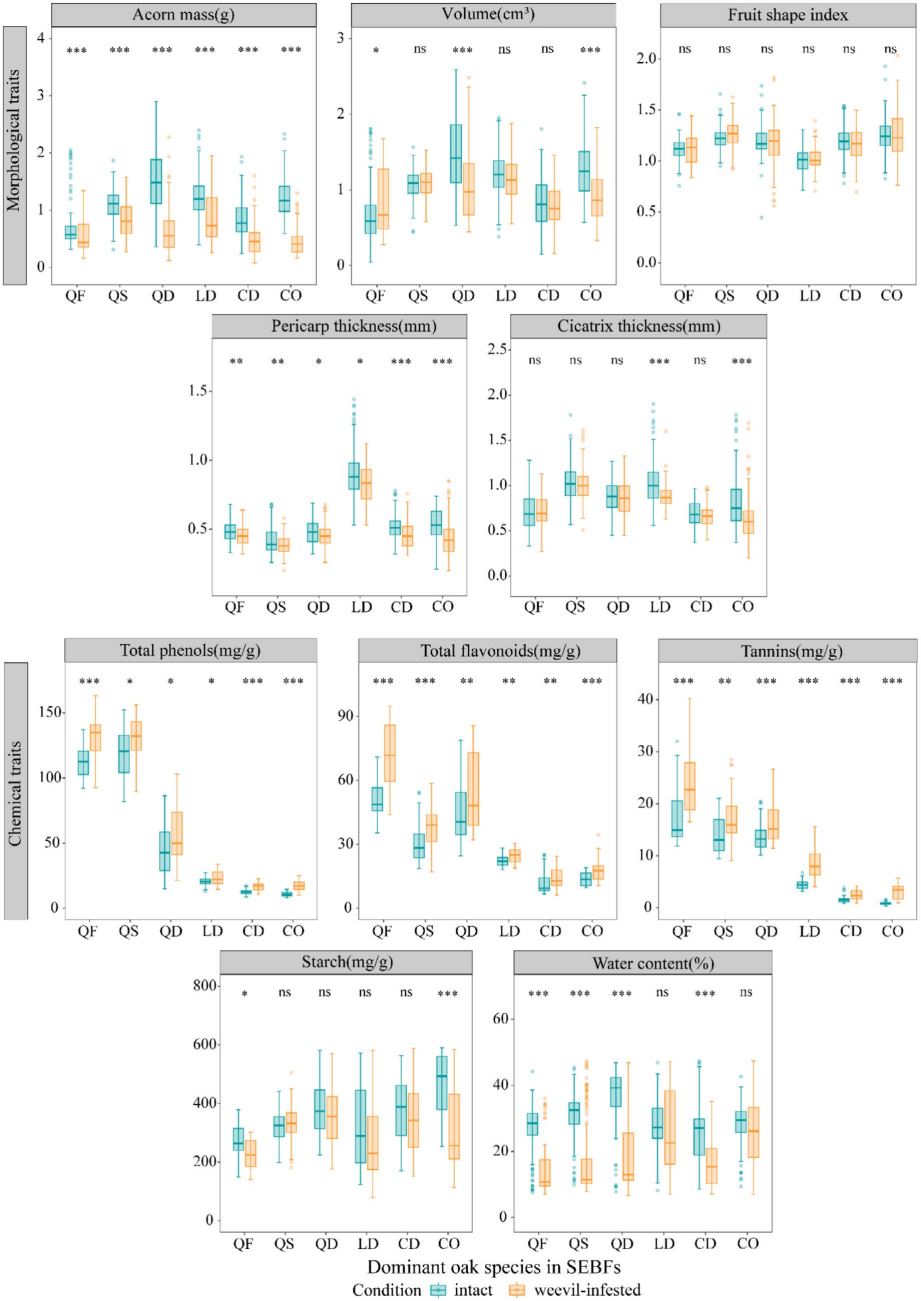
The differences between intact and weevil‐infested acorns in morphological and chemical traits. ‘ns’ at the top of the box plot means that there is no significant difference between intact and insect‐infested acorns, and ‘*’ means that there is a significant difference between groups. *p < 0.05; **p < 0.01; ***p < 0.001.

Comparative analysis between intact and infested acorns revealed significant trait modifications across all study species (Figure 6, Table S8). Morphologically, infested acorns exhibited universally reduced pericarp thickness (*p* < 0.001), with significant decreases in cicatrix thickness observed only in *L. dealbatus* (13.0% reduction) and *C. orthacantha* (20.0% reduction). Chemically, all dominant oak species showed significantly elevated secondary metabolite concentrations in infested acorns (*p* < 0.05). Nutrient storage displayed species‐specific responses, with *C. orthacantha* demonstrating a 48.0% decrease in starch content (*p* < 0.001) and *Q. schottkyana* showing a 64.7% reduction in water content (*p* < 0.001). These response patterns suggest acorns may counteract infestation pressure through upregulation of chemical defences.

#### 3.2.3 Trade‐Off Relationships of AFTs

**FIGURE 7:**
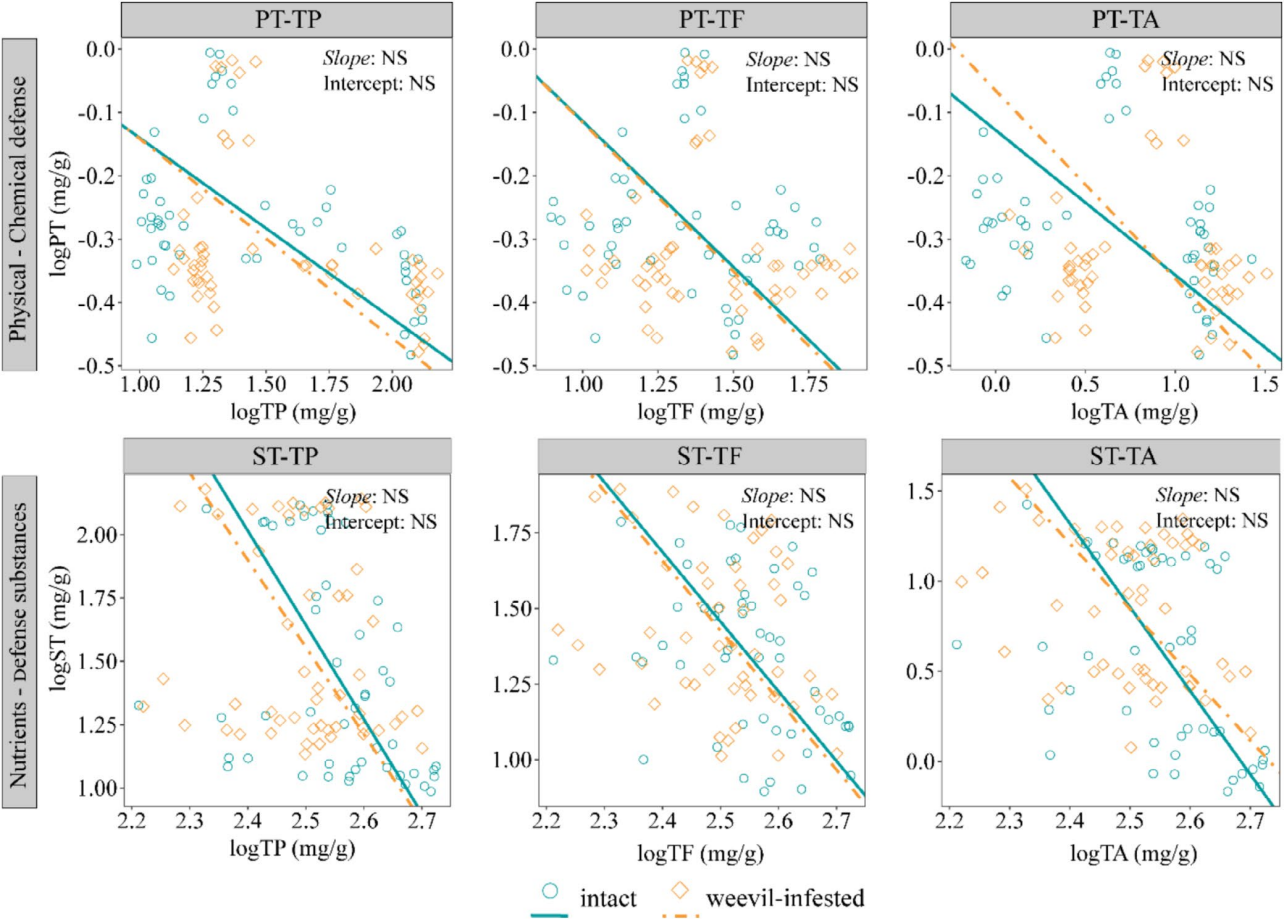
Trade‐off growth relationships between acorn functional traits of intact and weevil‐infested acorns of dominant oak species in SEBFs. When the slopes and intercept are not significantly different from each other, it indicates that there is an equal growth relationship between the tested factors. PT, pericarp thickness; ST, starch; TA, tannins; TF, total flavonoids; TP, total phenols.

Standardised major axis (SMA) regression analysis identified three consistent trade‐off patterns: (1) Significant negative correlations between physical (pericarp thickness) and chemical defences (common slope = −0.467 to −0.321, *p* < 0.001), indicating resource allocation trade‐offs; (2) Defence‐nutrient trade‐offs, particularly between tannins and starch (common slope = −4.191, *p* < 0.001); (3) Synergistic relationships among chemical defence compounds, evidenced by positive total phenols‐total flavonoids correlations (common slope = 1.545, *p* < 0.001) (Figure 7, Table S9). Crucially, the likelihood ratio tests confirmed no significant differences in these trade‐off patterns between intact and infested acorns (*p* > 0.05), demonstrating these represent inherent species‐specific resource allocation strategies rather than infestation‐induced responses.

## 4 Discussion

### 4.1 Interspecific Differentiation in AFTs and Defence Strategies

**FIGURE 8:**
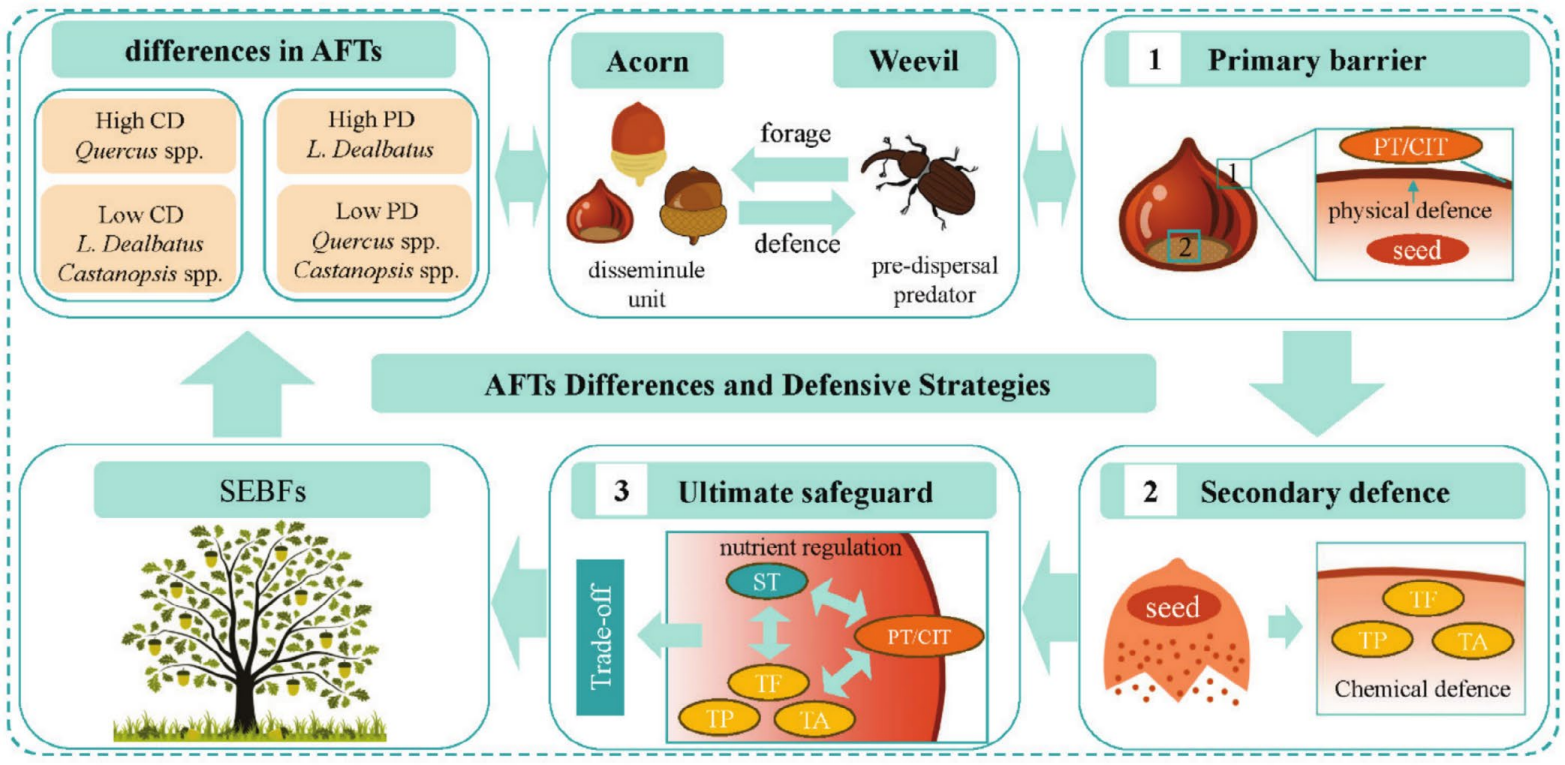
A graphic illustration of the effects and responses of weevil predation on acorns of dominant species in SEBFs. CD, chemical defence; CIT, cicatrix thickness; PD, physical defence; PT, pericarp thickness; ST, starch; TA, tannins; TF, total flavonoids; TP, total phenols. 1 and 2 represent the pericarp and cicatrix, respectively.

The six dominant oak species in SEBFs have developed complementary defence strategies through distinct combinations of AFTs. This differentiation reflects both species adaptation to heterogeneous environments and coevolution with acorn weevils (Muñoz et al. 2014; Bakis and Babaç 2015). Based on the results, we propose a comprehensive acorn defence strategy model (Figure 8). This model systematically explains: (1) a three‐tier defence system: physical defences form the primary barrier, chemical defences serve as secondary protection, and nutrient regulation (starch reallocation) constitutes the ultimate safeguard; (2) species‐specific strategies: *Quercus* species employ a ‘chemical specialisation’ strategy primarily relying on induced chemical defences, while *Lithocarpus* species adopt a ‘structural barrier’ strategy featuring physical defence plasticity, and *Castanopsis* species utilise a ‘nutrient buffering’ strategy characterised by high starch content with moderate defences; (3) these strategies are fundamentally constrained by plant resource allocation principles.

*L. dealbatus* employs an efficient physical defence system characterised by exceptionally thick pericarp (0.88 mm) and cicatrix (1.00 mm), which maintains infestation rates as low as 9%. This demonstrates the crucial role of mechanical barriers in preventing weevil damage. Notably, the strong negative correlation between cicatrix thickness and infestation rate highlights the ecological significance of this vulnerable structure, likely related to its anatomical features connecting acorns to branches. The cicatrix can be easily pierced by weevil mouthparts when it is not woody enough. This likely led to evolutionary pressure for thicker cicatrix walls as a defensive adaptation (Chen et al. 2020). Furthermore, variations in weevil species composition may reinforce this differentiation (Williams and Hawkins 2020; Fang et al. 2025). For instance, the stem‐boring weevil *Niphades castanea* was exclusively found in *L. dealbatus* acorns, suggesting that cicatrix thickening may represent an essential defensive adaptation against this specialised predator (Fang et al. 2025).

*Quercus* species relied more heavily on chemical defences, with significantly higher secondary metabolite concentrations than other species. These elevated levels of secondary compounds likely function through multiple mechanisms: on the one hand, they make acorns less tasty to weevils, thereby leading to a decrease in the species diversity of weevil predators (Shimada and Saitoh 2006; Onodera et al. 2017; Fang et al. 2025); on the other hand, they extend seed longevity through their antioxidant and antimicrobial functions (Coyotl‐Martinez et al. 2025). Chemical defences showed stronger explanatory power for the differentiation of interspecific overall functional strategies (Figure 4A–D), highlighting their importance as key functional traits in species coexistence. Although the results show conflicting relationships between total phenols and tannin content with infestation rates, this may reflect differences in weevil sensitivity to secondary metabolites, leading to varied selection patterns (Balaky et al. 2021; Stump et al. 2024). Our research on acorn weevil diversity also shows that there are differences in the species of weevil predators among these six oak species (Fang et al. 2025). Future research needs to further evaluate the mechanisms underlying weevil predators' sensitivity to secondary metabolites in acorns. Notably, despite having the highest tannin content (14.92 mg/g), *Q. franchetii* still showed 51% infestation rates, possibly because its smaller seed size (0.57 g) limited the effectiveness of chemical defences—consistent with the seed size‐defence trade‐off hypothesis (Bartlow et al. 2018; Chen, Antonelli, et al. 2025; Chen, Li, and Li 2025).

*Castanopsis* species exhibited an intermediate strategy, with moderate levels of both physical and chemical defences but the highest starch content (*C. orthacantha* reaching 493.13 mg/g). This ‘nutritional buffering’ strategy, combining high nutrition with moderate defences, may attract specialist weevils like *Pimelocerus perforatus*, maintaining relatively high infestation rates (Peguero et al. 2017; Liu et al. 2024; Fang et al. 2025). However, it may also create a ‘resource dilution’ effect, where weevils spread their eggs among numerous nutritious acorns, reducing damage to individual seeds (Mezquida et al. 2021).

These results collectively demonstrate that morphological and chemical traits play distinct yet complementary roles: morphological traits primarily account for variations in infestation rates, likely due to their function as direct physical barriers against weevil invasion; in contrast, chemical traits drive interspecific functional divergence as they represent key markers of adaptive strategies across different genera (Steele et al. 2021; Chen, Antonelli, et al. 2025; Chen, Li, and Li 2025). This functional partitioning constitutes a core feature of acorn defence strategies in response to biotic pressures.

### 4.2 Ecological Adaptation of Intraspecific AFTs Variation

While interspecific differences were significant, intraspecific variation in functional traits also affected weevil selection patterns. Continuous variation in AFTs within species created dynamic defence networks that influenced weevil foraging choices. Our study showed at the interspecific level, acorn volume was significantly positively correlated with infestation rate, while at the intraspecific level, starch content in *C. delavayi* positively correlated with infestation rates (*R* = 0.54), matching predictions from optimal foraging theory—female weevils preferred more nutritious acorns to improve offspring fitness (Pyke 1984; Koenig and Benedict 2002). However, this selective pressure may drive counter‐adaptation: populations with higher starch content showed stronger negative correlations with chemical defences like tannins (*R* = −0.73), indicating ‘nutrition‐defence’ trade‐offs also occur at microevolutionary scales (Zaret et al. 2024). Physical defence variation within species proved particularly important. *L. dealbatus* showed 17.56% variation in pericarp thickness, with infestation rates negatively correlated (*R* = −0.88) more strongly than interspecific differences. This suggests weevil mouthpart size and bite force may set an upper limit (Hughes and Vogler 2004; Iseki et al. 2011; Rühr et al. 2024), supporting ‘arms race’ theory where host defences and parasite feeding structures coevolve (Toju 2011).

Insects‐induced chemical defence responses demonstrated phenotypic plasticity's importance (Wang et al. 2023). All species increased secondary metabolites after infestation (Figure 6); though *Quercus* species showed smaller responses, possibly due to their already high baseline defences following the law of diminishing defence returns (Conde et al. 1998; Ramírez‐Valiente and Cavender‐Bares 2017). This plastic chemical defence represents an adaptive strategy developed through long‐term evolution, helping maintain population stability in complex environments (Karban et al. 1997; Mertens et al. 2021). Similar patterns occur in tobacco‐hornworm systems, where different tobacco varieties show varying induction strengths after herbivory (Kessler and Baldwin 2002). Acorn chemical defences may activate through jasmonic acid signalling pathways; though molecular mechanisms require further study (Wang et al. 2021).

### 4.3 Evolutionary Stability of Defence Trade‐Offs and Community‐Level Effects

This study revealed multiple trade‐off relationships in acorn defence systems and their ecological effects through standardised major axis (SMA) analysis. The physical–chemical defence trade‐off manifests as a significant negative correlation between them, and this relationship remains unaffected by infestation status, confirming it as a species‐specific inherent resource allocation strategy (Eichenberg et al. 2015; Wang et al. 2016). The simultaneously discovered defence‐nutrient trade‐offs and secondary metabolite synergies collectively form a multidimensional defence regulation network, where chemical defence substances achieve coordinated expression through shared biosynthetic pathways, while the negative correlation with nutrients reflects the classical ‘growth‐defence’ balance (Laughlin 2014a, 2014b; Agrawal 2020; Zaret et al. 2024). These evolutionarily stable trade‐off mechanisms shape the dynamic balance of the community: highly defence‐specialised *Quercus* species (e.g., *Q. schottkyana*) achieve relatively low infestation rates by investing substantial resources to build chemical defence systems, while *Castanopsis* species (e.g., *C. orthacantha*) adopt a ‘nutritional buffering strategy’ focusing on high nutrition (starch content 493.13 mg/g). Although enduring higher predation pressure, they can compensate for fitness loss through reproductive output (Mark 1993). Such complementary survival strategies create dynamic balance at the community level—when weevil populations concentrate attacks on one dominant species, other species gain competitive release, thereby maintaining system stability (Espelta, Bonal, and Sánchez‐Humanes 2009; Espelta, Cortés, et al. 2009; Espelta et al. 2017).

It is noteworthy that although these trade‐off relationships possess evolutionary stability, the explanatory power of functional traits for infestation rates remains relatively limited (*R* ^2^ = 0.38). This reflects, on one hand, the complexity of plant‐insect interactions, potentially involving acorn production (Bogdziewicz et al. 2018), unmeasured volatile signalling substances (Xu et al. 2023) or historical contingency events (Hoy et al. 2024). Although research on acorn production of dominant species in SEBFs remains scarce, observations during our sampling, actual sampling quantities (Table S1), and previous studies on other acorn species indicate that significant availability differences exist among different acorn taxa (Xiao et al. 2007; Koenig et al. 2013; Wang et al. 2015; Greenberg and Zarnoch 2018). For instance, *Quercus* species were more readily sampled (larger sample sizes) than *Lithocarpus* and *Castanopsis* species, a pattern consistent with broader evidence that production drives resource accessibility (Koenig et al. 2013). Such differences may influence insect foraging preferences—species with higher accessibility (likely linked to greater production) are more likely to become targets of concentrated insect feeding, whereas less accessible (low‐production) species face reduced predation pressure (Greenberg and Zarnoch 2018). Ultimately, interspecific differences in acorn production among these oaks and their impacts on infestation rates require further research. On the other hand, it suggests that in forest management, beyond focusing on key defence traits, we must comprehensively consider habitat heterogeneity and interspecies interaction networks (Parker et al. 2021; García‐Hernández et al. 2023). For example, protecting species with extreme defence strategies (e.g., the physically defensive *L. dealbatus*) can serve as ecological insurance, while maintaining functional group diversity can enhance community resilience against pest damage (Aleksandrov and Tonchev 2024). Future research should be multidimensional, integrating genomics, ecological modelling and long‐term field experiments to elucidate the dynamic interplay mechanisms between plant defence and insect feeding behaviour.

## 5 Conclusions

Our study reveals distinct acorn functional trait variations and weevil resistance strategies among six dominant oak species in semi‐humid evergreen broad‐leaved forests. *Lithocarpus dealbatus* exhibits the lowest infestation rate due to its thickest pericarp (physical defence), while *Quercus* species with chemical defences show higher infestation rates, and *Castanopsis* species adopt an intermediate strategy combining moderate defences with high starch content. Results demonstrate that pericarp thickness and tannin content are key traits influencing infestation rates, both showing significant negative correlations. Furthermore, infested acorns enhance defence by increasing secondary metabolites, with stable trade‐offs existing between physical and chemical defences, as well as between defence and nutrient allocation. These complementary strategies (physical barriers, chemical specialisation and nutrient regulation) collectively maintain forest community stability. The findings advance understanding of plant–animal coevolution and provide theoretical support for forest conservation. Future research should investigate molecular regulation of defence traits and environmental impacts on these interactions.

## Author Contributions

**Shengquan Fang:** conceptualization (equal), data curation (equal), investigation (lead), methodology (equal), writing – original draft (lead), writing – review and editing (lead). **Chongyun Wang:** conceptualization (lead), data curation (equal), methodology (lead), software (equal), writing – original draft (equal), writing – review and editing (equal). **Shaoji Hu:** data curation (equal), methodology (equal), writing – original draft (equal), writing – review and editing (equal). **Mingchun Peng:** data curation (equal), methodology (equal), writing – original draft (equal), writing – review and editing (equal). **Yongping Li:** data curation (equal), methodology (equal), writing – original draft (equal), writing – review and editing (equal). **Chunyan Lan:** data curation (equal), investigation (equal), software (equal). **Xinrong Li:** data curation (equal), investigation (equal), software (equal). **Dengpeng Chen:** data curation (equal), investigation (equal). **Biao Zhao:** data curation (equal), investigation (equal).

## Conflicts of Interest

The authors declare no conflicts of interest.

## Supporting information


**Data S1:** ece372045‐sup‐0001‐DataS1.zip.

## Data Availability

The data that support the findings of this study are uploaded as Supporting Information S1.
