# Supplementary material for: Acorn‐Weevil Interactions in Semi‐Humid Evergreen Broad‐Leaved Forests in Yunnan, China: Trade‐Offs Among Acorn Functional Traits
Source: Ecol Evol. 2025 Aug 21;15(8):e72045. doi: 10.1002/ece3.72045 (PMC12371126; doi:10.1002/ece3.72045)
Supplement: Supplementary file 1 — Data S1: ece372045‐sup‐0001‐DataS1.zip. [file ECE3-15-e72045-s001.zip › ece372045-sup-0002-FigureS1.docx]

**Figure S1.** Examples of the four states of acorns. a is *Q. schottkyana*, in sound condition; b is *C. delavayi*, weevil-infested; c is *Q. delavayi*, mildew; d is *C. orthacantha*, aborted.

**Figure S2.** Intact and weevil-infested acorns in SEBFs. Different letters represent abbreviations of species. QS - *Quercus schottkyana*, QD - *Q. delavayi*, QF - *Q. franchetii*, LD - *Lithocarpus dealbatus*, CD - *Castanopsis delavayi* and CO - *C. orthacantha*. Different number represent acorn status. 1 - acorn outsides; 2 - have insect holes in the pericarp or cicatrix of individual acorns; 3, 4, and 5 - acorns infested with weevils at different stages. All scales under the seed represent 1 mm.

**Figure S3.** Examples of morphometric measurements of acorns. Examples in the figure include: Pericarp thickness, Seed coat thickness, Cicatrix thickness, Longitudinal diameter, Transverse diameter.


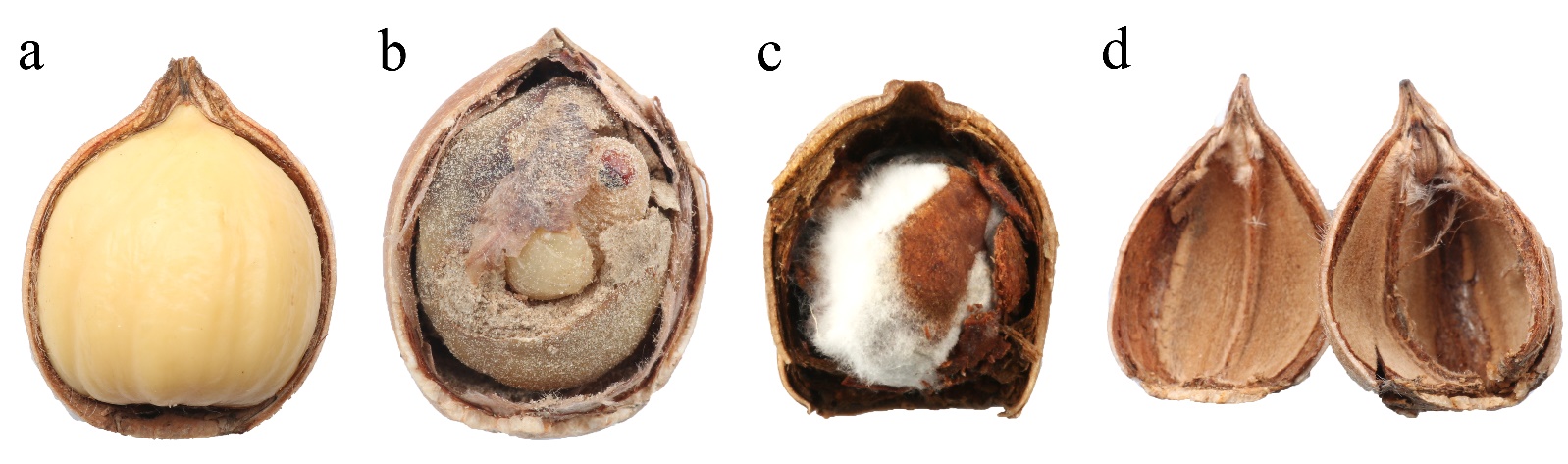


**Figure S1.** Examples of the four states of acorns. a is *Q. schottkyana*, in sound condition; b is *C. delavayi*, weevil-infested; c is *Q. delavayi*, mildew; d is *C. orthacantha*, aborted.


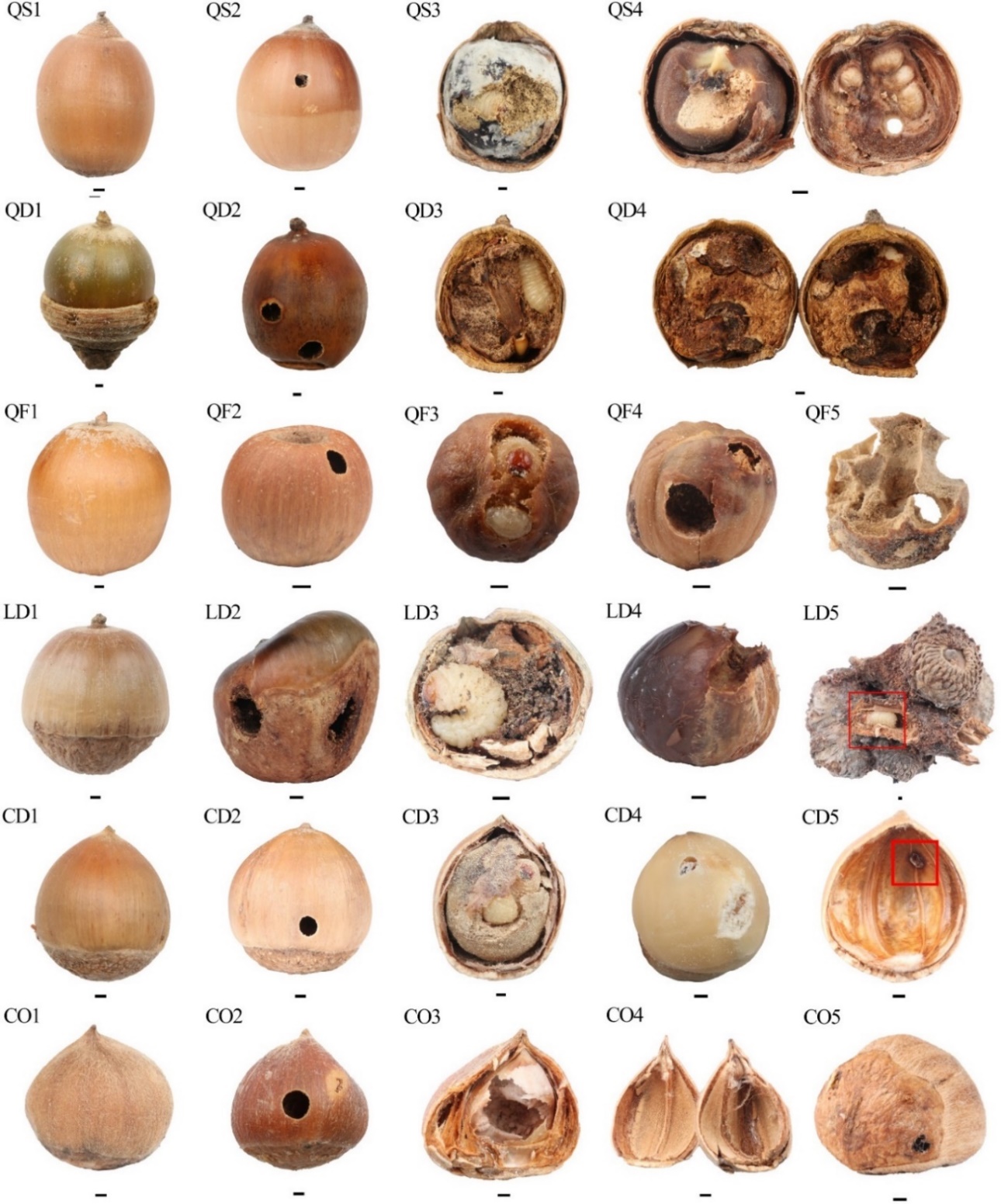


**Figure S2.** Intact and weevil-infested acorns in SEBFs. Different letters represent abbreviations of species. QS - *Quercus schottkyana*, QD - *Q. delavayi*, QF - *Q. franchetii*, LD - *Lithocarpus dealbatus*, CD - *Castanopsis delavayi* and CO - *C. orthacantha*. Different number represent acorn status. 1 - acorn outsides; 2 - have insect holes in the pericarp or cicatrix of individual acorns; 3, 4, and 5 - acorns infested with weevils at different stages. All scales under the seed represent 1 mm.


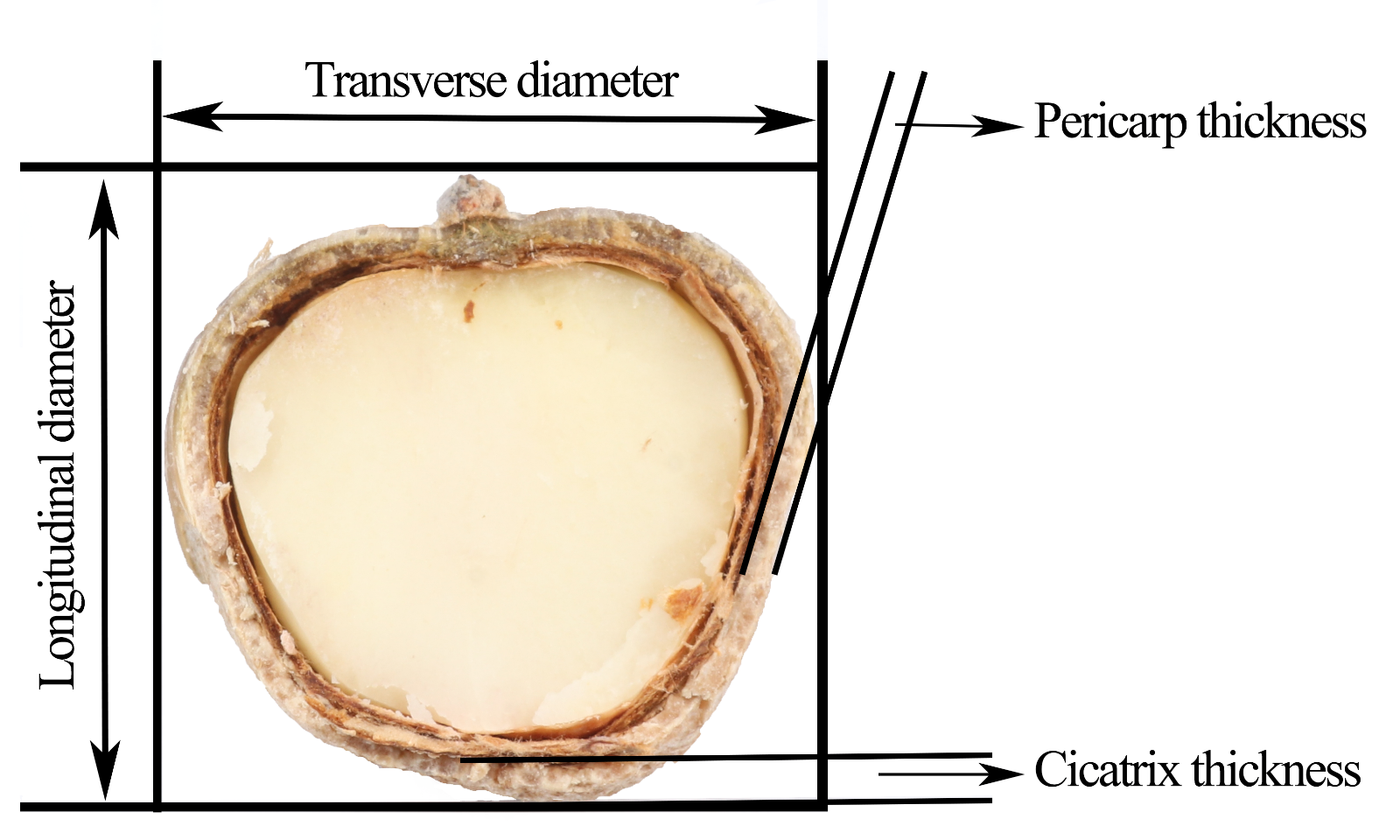


**Figure S3.** Examples of morphometric measurements of acorns. Examples in the figure include: Pericarp thickness, Seed coat thickness, Cicatrix thickness, Longitudinal diameter, Transverse diameter.
