# Supplementary material for: Acorn‐Weevil Interactions in Semi‐Humid Evergreen Broad‐Leaved Forests in Yunnan, China: Trade‐Offs Among Acorn Functional Traits
Source: Ecol Evol. 2025 Aug 21;15(8):e72045. doi: 10.1002/ece3.72045 (PMC12371126; doi:10.1002/ece3.72045)
Supplement: Supplementary file 1 — Data S1: ece372045‐sup‐0001‐DataS1.zip. [file ECE3-15-e72045-s001.zip › ece372045-sup-0003-TableS1.docx]

**Table S1.** Sampling sites and counts of acorns the six dominant oak species of semi-humid evergreen broad-leaved forests.

**Table S2.** The volume fitting formula for acorns of different family Fagaceae constructive species in SEBFs.

**Table S3.** Examination and analysis of differences in the state of different acorns.

**Table S4.** Differences in morphological and chemical traits of acorns of different Fagaceae species in SEBFs.

**Table S5.** Principal component analysis of functional traits of acorns of different family Fagaceae species in SEBFs.

**Table S6.** Coefficient estimates for each explaining variable and their corresponding p-values for functional traits of acorns tested by mixed-effects modeling.

**Table S7.** The Sperman correlation analysis between acorn infestation rate and AFTs in SEBFs.

**Table S8** The differences between intact and weevil-infested acorns in morphological and chemical traits.

**Table S9.** Results of standardized major axis estimation for the acorn functional traits of different family Fagaceae species.

**Table S1.** Sampling sites and counts of acorns the six dominant oak species of semi-humid evergreen broad-leaved forests.

| No. | Sampling location | Longitude | Latitude | Individuals | Total |
| --- | --- | --- | --- | --- | --- |
| *Castanopsis delavayi* | | | | | |
| CD-1 | Shizong, Qujing | 104.13115 | 24.595535 | 163 | 3367 |
| CD-2 | Chengjiang, Yuxi | 102.952614 | 24.511573 | 522 |  |
| CD-3 | Yongping, Dali | 99.655408 | 25.49154 | 315 |  |
| CD-4 | Yuanjiang, Yuxi | 101.981745 | 23.534923 | 720 |  |
| CD-5 | Eshan, Yuxi | 102.288451 | 24.160498 | 191 |  |
| CD-6 | Binchuan, Dali | 100.387166 | 25.961439 | 312 |  |
| CD-7 | Xishan, Kunming | 102.629021 | 24.969602 | 409 |  |
| CD-8 | Huaning, Yuxi | 102.882753 | 24.235641 | 244 |  |
| CD-9 | Xinping, Yuxi | 101.941568 | 23.970779 | 69 |  |
| CD-10 | Jianchuan, Dali | 99.873904 | 26.427872 | 118 |  |
| CD-11 | Binchuan, Dali | 100.908125 | 25.942347 | 37 |  |
| CD-12 | Yaoan, Chuxiong | 101.104273 | 25.641254 | 52 |  |
| CD-13 | Yaoan, Chuxiong | 101.054778 | 25.616378 | 215 |  |
| *Castanopsis orthacantha* | | | | | |
| CO-1 | Huize, Qujing | 103.80881 | 26.468238 | 49 | 1278 |
| CO-2 | Shizong, Qujing | 104.029918 | 24.700064 | 116 |  |
| CO-3 | Shizong, Qujing | 103.516541 | 25.188376 | 10 |  |
| CO-4 | Zhanyi, Qujing | 103.927056 | 25.90704 | 172 |  |
| CO-5 | Eshan, Yuxi | 102.286848 | 24.16035 | 248 |  |
| CO-6 | Xinping, Yuxi | 101.940652 | 23.972165 | 232 |  |
| CO-7 | Xishan, Kunming | 102.617525 | 25.070335 | 92 |  |
| CO-8 | Xundian, Kunming | 103.222804 | 25.6546 | 71 |  |
| CO-9 | Weishan, Dali | 100.350131 | 25.178924 | 60 |  |
| CO-10 | Chuxiong, Chuxiong | 101.406044 | 25.024057 | 72 |  |
| CO-11 | Mouding, Chuxiong | 101.422103 | 25.312822 | 31 |  |
| CO-12 | Yaoan, Chuxiong | 101.087907 | 25.617267 | 125 |  |
| *Lithocarpus dealbatus* | | | | | |
| LD-1 | Shizong, Qujing | 104.002002 | 24.641172 | 492 | 3367 |
| LD-2 | Luoping, Qujing | 104.183118 | 25.099587 | 500 |  |
| LD-3 | Xinping, Yuxi | 101.941568 | 23.970779 | 340 |  |
| LD-4 | Xishan, Kunming | 102.631395 | 24.9592972 | 88 |  |
| LD-5 | Xishan, Kunming | 102.617525 | 25.070335 | 433 |  |
| LD-6 | Xishan, Kunming | 102.575142 | 24.840145 | 265 |  |
| LD-7 | Panlong, Kunming | 102.783597 | 25.349328 | 92 |  |
| LD-8 | Dayao, Chuxiong | 101.233213 | 25.953357 | 97 |  |
| LD-9 | Yaoan, Chuxiong | 101.12126 | 25.629101 | 900 |  |
| LD-10 | Yaoan, Chuxiong | 101.090898 | 25.614779 | 160 |  |
| *Quercus delavayi* | | | | | |
| QD-1 | Shizong, Qujing | 103.539404 | 25.258308 | 59 | 2442 |
| QD-2 | Xishan, Kunming | 102.65539 | 24.808328 | 354 |  |
| QD-3 | Yaoan, Chuxiong | 101.104983 | 25.641275 | 418 |  |
| QD-4 | Luquan, Kunming | 102.691412 | 25.680281 | 286 |  |
| QD-5 | Dayao, Chuxiong | 101.360534 | 25.81331 | 186 |  |
| QD-6 | Jianchuan, Dali | 99.826294 | 26.285992 | 167 |  |
| QD-7 | Anning, Kunming | 102.299012 | 24.93265 | 30 |  |
| QD-8 | Luquan, Kunming | 102.379057 | 25.727054 | 575 |  |
| QD-9 | Weishan, Dali | 100.216362 | 25.187592 | 55 |  |
| QD-10 | Yaoan, Chuxiong | 101.093859 | 25.61401 | 312 |  |
| *Quercus schottkyana* | | | | | |
| QS-1 | Shizong, Qujing | 104.129988 | 24.589039 | 248 | 5139 |
| QS-2 | Shizong, Qujing | 103.825917 | 24.771377 | 583 |  |
| QS-3 | Xishan, Kunming | 102.592692 | 24.846605 | 460 |  |
| QS-4 | Xishan, Kunming | 102.629153 | 24.962467 | 485 |  |
| QS-5 | Binchuan, Dali | 100.3731 | 25.958716 | 707 |  |
| QS-6 | Yuanjiang, Yuxi | 102.06713 | 23.665909 | 522 |  |
| QS-7 | Yaoan, Chuxiong | 101.291904 | 25.466706 | 719 |  |
| QS-8 | Lufeng, Chuxiong | 101.790564 | 25.109569 | 571 |  |
| QS-9 | Binchuan, Dali | 100.908125 | 25.942347 | 231 |  |
| QS-10 | Yongren, Chuxiong | 101.799374 | 26.159017 | 613 |  |
| *Quercus franchetii* | | | | | |
| QF-1 | Lufeng, Chuxiong | 101.779314 | 25.109572 | 1323 | 2292 |
| QF-2 | Binchuan, Dali | 100.402485 | 25.90758 | 216 |  |
| QF-3 | Eshan, Yuxi | 102.39629 | 24.160946 | 331 |  |
| QF-4 | Xishan, Kunming | 102.604178 | 24.846233 | 322 |  |
| QF-5 | Yongren, Chuxiong | 101.802796 | 26.158986 | 100 |  |

**Table S1.** (continued)

**Table S2.** The volume fitting formula for acorns of different family Fagaceae constructive species in SEBFs.

| Host plant | The fitting formula of volumn | R^2^ |
| --- | --- | --- |
| *Quercus franchetii* | V=0.101*L-0.024*W | 0.929 |
| *Quercus schottkyana* | V=-0.029*L+0.125*W | 0.989 |
| *Quercus delavayi* | V=-0.115*L+0.259*W | 0.983 |
| *Lithocarpus dealbatus* | V=0.014*L+0.073*W | 0.989 |
| *Castanopsis delavayi* | V=-0.015*L+0.091*W | 0.959 |
| *Castanopsis orthacantha* | V=-0.021*L+0.137*W | 0.960 |

**Table S3.** Examination and analysis of differences in the status of different acorns.

| Status | Species | Median (IQR) | CV | *H* | *df* | *P* |
| --- | --- | --- | --- | --- | --- | --- |
| Intact | CD | 38.0(23.0, 55.5) | 56.07 | 26.01 | 5 | 0.000*** |
|  | CO | 16.0(6.3, 19.0) | 71.86 |  |  |  |
|  | LD | 70.0(69.5, 72.5) | 10.06 |  |  |  |
|  | QD | 8.0(5.5, 27.0) | 113.12 |  |  |  |
|  | QF | 41.0(33.5, 48.5) | 22.71 |  |  |  |
|  | QS | 40.0(22.0, 56.0) | 52.78 |  |  |  |
| Weevil-infested | CD | 18.0(16.8, 29.0) | 46.98 | 22.66 | 5 | 0.000*** |
|  | CO | 28.0(19.0, 38.7) | 57.43 |  |  |  |
|  | LD | 9.0(6.0, 14.0) | 68.03 |  |  |  |
|  | QD | 50.0(28.0, 53.0) | 41.92 |  |  |  |
|  | QF | 51.0(45.5, 57.0) | 15.00 |  |  |  |
|  | QS | 34.0(24.0, 46.0) | 47.67 |  |  |  |
| Mildew | CD | 4.0(1.0, 7.0) | 84.42 | 10.65 | 5 | 0.060 |
|  | CO | 2.0(0.0, 4.0) | 93.57 |  |  |  |
|  | LD | 1.0(0.0, 4.5) | 120.55 |  |  |  |
|  | QD | 10.0(5.5, 16.5) | 68.11 |  |  |  |
|  | QF | 1.0(0.0, 2.5) | 127.66 |  |  |  |
|  | QS | 2.0(0.0, 4.0) | 125.08 |  |  |  |
| Aborted | CD | 32.0(22.3, 51.0) | 64.16 | 23.24 | 5 | 0.000*** |
|  | CO | 50.0(46.0, 59.0) | 25.39 |  |  |  |
|  | LD | 17.0(14.5, 19.5) | 54.36 |  |  |  |
|  | QD | 24.0(17.5, 28.5) | 71.73 |  |  |  |
|  | QF | 6.0(5.5, 6.5) | 27.22 |  |  |  |
|  | QS | 16.0(8.0, 22.0) | 90.28 |  |  |  |

Note: *P* > 0.05: no difference; *p* < 0.001: * * *, there is a very significant difference.

**Table S4.** Differences in morphological and chemical traits of acorns of different Fagaceae species in SEBFs.

| Functional traits | Species | Sample count | Median (IQR) | CV (%) | *H* | *P-*value |
| --- | --- | --- | --- | --- | --- | --- |
| Morphological traits |  |  |  |  |  |  |
| Acorn mass (g) | QF | 112 | 0.57(0.50, 0.72) e | 56.47 | 308.39 | 0.000*** |
|  | QS | 186 | 1.12(0.93, 1.26) c | 22.60 |  |  |
|  | QD | 88 | 1.49(1.12, 1.88) a | 38.90 |  |  |
|  | LD | 278 | 1.20(1.01, 1.42) b | 26.39 |  |  |
|  | CD | 229 | 0.77(0.62, 1.04) d | 37.36 |  |  |
|  | CO | 83 | 1.17(0.98, 1.42) bc | 30.05 |  |  |
| Volume (cm^3^) | QF | 112 | 0.59(0.42, 0.80) e | 57.47 | 316.86 | 0.000*** |
|  | QS | 186 | 1.09(0.95, 1.20) c | 18.44 |  |  |
|  | QD | 88 | 1.42(1.09, 1.86) a | 36.16 |  |  |
|  | LD | 278 | 1.20(1.04, 1.39) b | 22.52 |  |  |
|  | CD | 229 | 0.81(0.58, 1.07) d | 38.54 |  |  |
|  | CO | 83 | 1.25(0.99, 1.5) ab | 30.46 |  |  |
| Fruit shape index | QF | 112 | 1.12(1.05, 1.18) d | 10.45 | 338.80 | 0.000*** |
|  | QS | 186 | 1.22(1.16, 1.28) ab | 8.62 |  |  |
|  | QD | 88 | 1.17(1.12, 1.27) bc | 13.69 |  |  |
|  | LD | 278 | 1.01(0.92, 1.08) e | 12.83 |  |  |
|  | CD | 229 | 1.19(1.11, 1.28) c | 11.55 |  |  |
|  | CO | 83 | 1.24(1.15, 1.34) a | 13.78 |  |  |
| Pericarp thickness (mm) | QF | 112 | 0.48(0.43, 0.53) d | 14.24 | 653.82 | 0.000*** |
|  | QS | 186 | 0.39(0.35, 0.48) e | 20.94 |  |  |
|  | QD | 88 | 0.48(0.41, 0.54) cd | 18.25 |  |  |
|  | LD | 278 | 0.88(0.79, 0.98) a | 17.56 |  |  |
|  | CD | 229 | 0.51(0.46, 0.56) bc | 17.03 |  |  |
|  | CO | 83 | 0.53(0.46, 0.63) b | 20.73 |  |  |
| Cicatrix thickness (mm) | QF | 112 | 0.69(0.56, 0.85) d | 29.47 | 371.84 | 0.000*** |
|  | QS | 186 | 1.02(0.89, 1.15) a | 20.81 |  |  |
|  | QD | 88 | 0.88(0.76, 1.00) b | 19.71 |  |  |
|  | LD | 278 | 1.00(0.86, 1.15) a | 21.53 |  |  |
|  | CD | 229 | 0.68(0.59, 0.80) d | 20.30 |  |  |
|  | CO | 83 | 0.75(0.62, 0.96) c | 38.54 |  |  |
| Chemical traits | | | | | | |
| Water content (%) | QF | 112 | 28.95(25.20, 32.14) c | 23.20 | 147.47 | 0.000*** |
|  | QS | 186 | 32.52(28.22, 34.78) b | 21.23 |  |  |
|  | QD | 88 | 39.28(33.70, 42.89) a | 28.12 |  |  |
|  | LD | 278 | 27.26(23.97, 33.13) c | 22.26 |  |  |
|  | CD | 229 | 26.96(19.19, 29.77) d | 32.24 |  |  |
|  | CO | 83 | 29.50(25.76, 32.20) e | 38.56 |  |  |
| Total phenols (mg/g) | QF | 20 | 112.64(92.98, 138.66) a | 29.33 | 218.90 | 0.000*** |
|  | QS | 45 | 120.59(104.11, 132.91) a | 15.44 |  |  |
|  | QD | 45 | 42.72(28.78, 58.48) b | 50.96 |  |  |
|  | LD | 40 | 20.46(18.79, 22.44) c | 16.38 |  |  |
|  | CD | 55 | 12.50(11.19, 13.57) d | 16.12 |  |  |
|  | CO | 40 | 10.54(9.37, 12.23) d | 15.28 |  |  |
| Total flavonoids (mg/g) | QF | 20 | 48.70(45.64, 56.48) a | 17.75 | 187.15 | 0.000*** |
|  | QS | 45 | 28.33(23.53, 34.85) b | 32.26 |  |  |
|  | QD | 45 | 40.55(32.04, 54.40) a | 43.63 |  |  |
|  | LD | 40 | 22.00(20.28, 23.78) c | 11.46 |  |  |
|  | CD | 55 | 9.41(8.24, 14.30) d | 45.81 |  |  |
|  | CO | 40 | 13.53(10.66, 16.64) d | 23.32 |  |  |
| Tannins (mg/g) | QF | 20 | 14.92(13.69, 20.56) a | 32.87 | 208.24 | 0.000*** |
|  | QS | 45 | 13.01(10.98, 16.96) a | 24.74 |  |  |
|  | QD | 45 | 13.22(11.68, 14.88) a | 24.56 |  |  |
|  | LD | 40 | 4.40(3.76, 4.91) b | 19.87 |  |  |
|  | CD | 55 | 1.51(1.12, 1.75) c | 41.58 |  |  |
|  | CO | 40 | 1.63(1.29, 1.75) c | 23.64 |  |  |
| Starch (mg/g) | QF | 20 | 263.77(239.52, 315.20) c | 28.96 | 55.14 | 0.000*** |
|  | QS | 45 | 325.13(287.15, 354.35) c | 15.08 |  |  |
|  | QD | 45 | 373.63(314.61, 446.67) b | 26.80 |  |  |
|  | LD | 40 | 289.78(197.60, 445.94) c | 44.28 |  |  |
|  | CD | 55 | 388.82(290.95, 461.57) b | 28.51 |  |  |
|  | CO | 40 | 493.13(379.33, 560.62) a | 22.83 |  |  |

Note: QF - *Quercus franchetii*, QS - *Q. schottkyana*, QD - *Q. delavayi*, LD - *Lithocarpus dealbatus*, CD - *Castanopsis delavayi*, CO - *C. orthacantha*. Different lowercase letters mean significant differences in AFTs between species. *** means that there is a highly significant difference between the species.

**Table S5.** Principal component analysis of functional traits of acorns of different family Fagaceae species in SEBFs.

| Functional traits | | Loadings | | |  | Contribution (%) | | |
| --- | --- | --- | --- | --- | --- | --- | --- | --- |
|  |  | PC1 | PC2 | PC3 |  | PC1 | PC2 | PC3 |
| Morphological traits | cicatrix thickness | 0.214 | 0.764 | 0.272 |  | 1.492 | 22.348 | 4.927 |
|  | fresh weight | -0.211 | 0.811 | 0.293 |  | 1.451 | 25.145 | 5.719 |
|  | fruit shape index | -0.115 | -0.491 | 0.697 |  | 0.428 | 9.219 | 32.359 |
|  | pericarp thickness | -0.353 | 0.617 | -0.550 |  | 4.074 | 14.541 | 20.156 |
|  | volume | -0.165 | 0.770 | 0.230 |  | 0.885 | 22.692 | 3.521 |
| Chemical traits | water content | -0.334 | 0.310 | 0.279 |  | 3.646 | 3.684 | 5.191 |
|  | starch | -0.468 | -0.107 | 0.592 |  | 7.152 | 0.434 | 23.305 |
|  | tannins | 0.947 | 0.138 | 0.091 |  | 29.283 | 0.730 | 0.553 |
|  | total flavonoids | 0.885 | 0.148 | -0.016 |  | 25.608 | 0.833 | 0.018 |
|  | total phenols | 0.892 | 0.099 | 0.253 |  | 25.981 | 0.374 | 4.250 |
|  | Eigenvalue |  |  |  |  | 3.026 | 2.615 | 1.503 |
|  | Variance  contribution rate/% |  |  |  |  | 30.614 | 26.148 | 15.030 |
|  | Accumulated variance  contribution rate/% |  |  |  |  | 30.614 | 56.762 | 71.792 |

**Table S6.** Coefficient estimates for each explaining variable and their corresponding p-values for functional traits of acorns tested by mixed-effects modeling.

| Functional traits | | Estimate | SE | *df* | *t* value | Pr(>\|t\|) |
| --- | --- | --- | --- | --- | --- | --- |
| Morphological traits | acorn mass | 0.30 | 0.12 | 145.77 | 2.52 | 0.013 |
|  | volume | 0.34 | 0.08 | 142.78 | 4.25 | 0.000 |
|  | pericarp thickness | -0.28 | 0.07 | 28.98 | -3.94 | 0.000 |
|  | cicatrix thickness | -0.02 | 0.06 | 243.65 | -0.38 | 0.706 |
|  | fruit shape index | -0.10 | 0.12 | 13.26 | -0.86 | 0.404 |
| Chemical traits | total phenols | 0.13 | 0.06 | 158.49 | 2.20 | 0.029 |
|  | total flavonoids | 0.08 | 0.10 | 38.57 | 0.76 | 0.451 |
|  | tannins | -0.27 | 0.08 | 238.70 | -3.46 | 0.001 |
|  | starch | 0.06 | 0.05 | 248.37 | 1.07 | 0.286 |
|  | water content | -0.04 | 0.05 | 254.13 | -0.75 | 0.456 |

**Table S7.** The Sperman correlation analysis between acorn infestation rate and AFTs in SEBFs.

|  | *C. delavayi* | |  | *C. orthacantha* | |  | *L. dealbatus* | |  | *Q. delavayi* | |  | *Q. franchetii* | |  | *Q. schottkyana* | |
| --- | --- | --- | --- | --- | --- | --- | --- | --- | --- | --- | --- | --- | --- | --- | --- | --- | --- |
|  | *R* | *P* |  | *R* | *P* |  | *R* | *P* |  | *R* | *P* |  | *R* | *P* |  | *R* | *P* |
| Morphological traits | | | | | | | | | | | | | | | | | |
| AM | 0.39 | 0.24 |  | -0.43 | 0.35 |  | -0.06 | 0.55 |  | -0.31 | 0.35 |  | 0.61 | 0.60 |  | 0.14 | 0.65 |
| VO | 0.59 | 0.17 |  | -0.45 | 0.14 |  | -0.44 | 0.38 |  | -0.21 | 0.35 |  | 0.66 | 0.60 |  | 0.27 | 0.51 |
| FSI | -0.12 | 0.65 |  | -0.25 | 0.48 |  | -0.01 | 0.61 |  | 0.05 | 0.84 |  | -0.08 | 0.80 |  | -0.32 | 0.98 |
| PT | **-0.68*** | 0.01 |  | **-0.57*** | 0.02 |  | **-0.88*** | 0.02 |  | **-0.73*** | 0.05 |  | -0.47 | 0.80 |  | -0.64 | 0.06 |
| CIT | 0.01 | 0.29 |  | **-0.83*** | 0.00 |  | **-0.71*** | 0.02 |  | -0.54 | 0.16 |  | -0.04 | 0.80 |  | -0.55 | 0.17 |
| Chemical traits | | | | | | | | | | | | | | | | | |
| TP | **-0.54*** | 0.02 |  | -0.58 | 0.12 |  | -0.67 | 0.08 |  | **-0.61*** | 0.05 |  | -0.57 | 0.40 |  | **-0.68*** | 0.04 |
| TF | -0.52 | 0.18 |  | **-0.72*** | 0.02 |  | **-0.68*** | 0.03 |  | **-0.71*** | 0.04 |  | -0.71 | 0.20 |  | **-0.68*** | 0.04 |
| TA | **-0.73*** | 0.02 |  | **-0.57*** | 0.03 |  | **-0.77*** | 0.00 |  | **-0.76*** | 0.00 |  | -0.68 | 0.60 |  | **-0.79*** | 0.00 |
| ST | **0.54*** | 0.02 |  | 0.47 | 0.29 |  | 0.45 | 0.14 |  | 0.58 | 0.23 |  | 0.52 | 0.40 |  | 0.47 | 0.14 |
| WC | 0.23 | 0.64 |  | 0.14 | 0.72 |  | 0.05 | 0.99 |  | 0.40 | 0.66 |  | -0.03 | 1.00 |  | -0.25 | 0.41 |

Note: AM - acorn mass; VO - volume; FSI - fruit shape index; PT - pericarp thickness; CIT - cicatrix thickness; TP - total phenols; TF - total flavonoids; TA - tannins: ST - starch; WC - water content. **P* < 0.05 significant difference.

**Table S8.** The differences between intact and weevil-infested acorns in morphological and chemical traits.

| AFTs | Species | Type | N | Median (IQR) | CV (%) | Median difference | Range of variation | Wilcox_stat | Wilcox_p |
| --- | --- | --- | --- | --- | --- | --- | --- | --- | --- |
| Morphological traits |  |  |  |  |  |  |  |  |  |
| Acorn mass (g) | CD | Intact | 229 | 0.77 (0.62, 1.04) | 36.88 | -0.32 | -0.42 | 19051 | 0.000 |
|  |  | Weevil-infested | 101 | 0.45 (0.27, 0.61) | 56.87 |  |  |  |  |
|  | CO | Intact | 83 | 1.17 (0.98, 1.42) | 30.05 | -0.76 | -0.65 | 12363 | 0.000 |
|  |  | Weevil-infested | 153 | 0.41 (0.27, 0.54) | 49.09 |  |  |  |  |
|  | LD | Intact | 278 | 1.20 (1.01, 1.42) | 26.39 | -0.47 | -0.39 | 9228.5 | 0.000 |
|  |  | Weevil-infested | 44 | 0.73 (0.54, 1.22) | 50.07 |  |  |  |  |
|  | QD | Intact | 88 | 1.49 (1.12, 1.88) | 38.90 | -0.93 | -0.63 | 13880.5 | 0.000 |
|  |  | Weevil-infested | 176 | 0.55 (0.35, 0.82) | 59.04 |  |  |  |  |
|  | QF | Intact | 112 | 0.57 (0.50, 0.72) | 56.47 | -0.14 | -0.24 | 5783 | 0.000 |
|  |  | Weevil-infested | 76 | 0.44 (0.36, 0.75) | 53.94 |  |  |  |  |
|  | QS | Intact | 186 | 1.12 (0.93, 1.26) | 22.60 | -0.31 | -0.28 | 22477.5 | 0.000 |
|  |  | Weevil-infested | 158 | 0.81 (0.59, 1.06) | 35.89 |  |  |  |  |
| Volume (cm^3^) | CD | Intact | 229 | 0.81 (0.58, 1.07) | 38.54 | -0.05 | -0.07 | 12366.5 | 0.316 |
|  |  | Weevil-infested | 101 | 0.75 (0.61, 0.98) | 36.06 |  |  |  |  |
|  | CO | Intact | 83 | 1.25 (0.99, 1.51) | 30.46 | -0.38 | -0.31 | 9593 | 0.000 |
|  |  | Weevil-infested | 153 | 0.86 (0.66, 1.14) | 38.07 |  |  |  |  |
|  | LD | Intact | 278 | 1.20 (1.04, 1.39) | 22.52 | -0.07 | -0.06 | 6805 | 0.230 |
|  |  | Weevil-infested | 44 | 1.13 (0.95, 1.34) | 24.60 |  |  |  |  |
|  | QD | Intact | 88 | 1.42 (1.09, 1.86) | 36.16 | -0.45 | -0.31 | 11086 | 0.000 |
|  |  | Weevil-infested | 176 | 0.97 (0.67, 1.35) | 43.54 |  |  |  |  |
|  | QF | Intact | 112 | 0.59 (0.42, 0.80) | 57.47 | 0.08 | 0.14 | 3447 | 0.027 |
|  |  | Weevil-infested | 76 | 0.67 (0.48, 1.28) | 52.76 |  |  |  |  |
|  | QS | Intact | 186 | 1.09 (0.95, 1.20) | 18.44 | 0.01 | 0.01 | 13810 | 0.336 |
|  |  | Weevil-infested | 158 | 1.10 (0.96, 1.22) | 17.97 |  |  |  |  |
| Fruit shape index | CD | Intact | 229 | 1.19 (1.11, 1.27) | 11.54 | -0.02 | -0.02 | 12678 | 0.163 |
|  |  | Weevil-infested | 101 | 1.17 (1.05, 1.28) | 14.10 |  |  |  |  |
|  | CO | Intact | 83 | 1.24 (1.15, 1.34) | 13.78 | -0.01 | -0.01 | 6499 | 0.766 |
|  |  | Weevil-infested | 153 | 1.23 (1.09, 1.42) | 18.63 |  |  |  |  |
|  | LD | Intact | 278 | 1.01 (0.92, 1.08) | 12.69 | -0.01 | -0.01 | 5809 | 0.593 |
|  |  | Weevil-infested | 44 | 1.00 (0.96, 1.09) | 12.94 |  |  |  |  |
|  | QD | Intact | 88 | 1.17 (1.12, 1.27) | 13.69 | 0.03 | 0.02 | 7874 | 0.825 |
|  |  | Weevil-infested | 176 | 1.20 (1.06, 1.30) | 16.51 |  |  |  |  |
|  | QF | Intact | 112 | 1.12 (1.05, 1.18) | 10.45 | 0.01 | 0.01 | 4166 | 0.807 |
|  |  | Weevil-infested | 76 | 1.13 (0.99, 1.22) | 13.41 |  |  |  |  |
|  | QS | Intact | 186 | 1.22 (1.16, 1.28) | 8.63 | 0.02 | 0.02 | 11453 | 0.423 |
|  |  | Weevil-infested | 158 | 1.24 (1.18, 1.32) | 10.31 |  |  |  |  |
| Pericarp thickness (mm) | CD | Intact | 229 | 0.51 (0.46, 0.56) | 17.03 | -0.06 | -0.12 | 15332.5 | 0.000 |
|  |  | Weevil-infested | 101 | 0.45 (0.38, 0.52) | 22.06 |  |  |  |  |
|  | CO | Intact | 83 | 0.53 (0.46, 0.63) | 20.73 | -0.11 | -0.21 | 9545.5 | 0.000 |
|  |  | Weevil-infested | 153 | 0.42 (0.34, 0.50) | 30.18 |  |  |  |  |
|  | LD | Intact | 278 | 0.88 (0.79, 0.98) | 17.56 | -0.05 | -0.05 | 7443 | 0.021 |
|  |  | Weevil-infested | 44 | 0.84 (0.72, 0.93) | 17.91 |  |  |  |  |
|  | QD | Intact | 88 | 0.48 (0.41, 0.54) | 18.25 | -0.03 | -0.06 | 9164.5 | 0.015 |
|  |  | Weevil-infested | 176 | 0.45 (0.40, 0.50) | 17.30 |  |  |  |  |
|  | QF | Intact | 112 | 0.48 (0.43, 0.53) | 14.24 | -0.03 | -0.06 | 5326 | 0.003 |
|  |  | Weevil-infested | 76 | 0.45 (0.40, 0.50) | 15.42 |  |  |  |  |
|  | QS | Intact | 186 | 0.39 (0.35, 0.48) | 20.94 | -0.01 | -0.03 | 17217 | 0.006 |
|  |  | Weevil-infested | 158 | 0.38 (0.34, 0.43) | 17.37 |  |  |  |  |
| Cicatrix thickness (mm) | CD | Intact | 229 | 0.68 (0.59, 0.80) | 20.30 | -0.02 | -0.03 | 12728 | 0.145 |
|  |  | Weevil-infested | 101 | 0.66 (0.58, 0.73) | 19.32 |  |  |  |  |
|  | CO | Intact | 83 | 0.75 (0.61, 0.96) | 38.71 | -0.15 | -0.20 | 8948 | 0.000 |
|  |  | Weevil-infested | 153 | 0.60 (0.47, 0.72) | 38.20 |  |  |  |  |
|  | LD | Intact | 278 | 1.00 (0.86, 1.15) | 21.53 | -0.13 | -0.13 | 8309.5 | 0.000 |
|  |  | Weevil-infested | 44 | 0.87 (0.80, 0.95) | 19.82 |  |  |  |  |
|  | QD | Intact | 88 | 0.88 (0.76, 1.00) | 19.82 | -0.02 | -0.02 | 8023 | 0.478 |
|  |  | Weevil-infested | 176 | 0.86 (0.72, 1.00) | 21.20 |  |  |  |  |
|  | QF | Intact | 112 | 0.69 (0.56, 0.85) | 29.47 | 0.00 | 0.01 | 3997.5 | 0.578 |
|  |  | Weevil-infested | 76 | 0.69 (0.61, 0.85) | 25.25 |  |  |  |  |
|  | QS | Intact | 186 | 1.02 (0.89, 1.15) | 20.81 | -0.02 | -0.02 | 15297 | 0.512 |
|  |  | Weevil-infested | 158 | 1.00 (0.89, 1.10) | 18.47 |  |  |  |  |
| Chemical traits |  |  |  |  |  |  |  |  |  |
| Total phenols (mg/g) | CD | Intact | 55 | 12.50 (11.19, 13.57) | 16.12 | 4.56 | 0.37 | 436.5 | 0.000 |
|  |  | Weevil-infested | 50 | 17.06 (14.29, 18.51) | 18.22 |  |  |  |  |
|  | CO | Intact | 40 | 10.54 (9.37, 12.23) | 15.28 | 6.59 | 0.63 | 124.5 | 0.000 |
|  |  | Weevil-infested | 40 | 17.12 (14.50, 20.00) | 23.54 |  |  |  |  |
|  | LD | Intact | 40 | 20.46 (18.79, 22.44) | 16.38 | 1.61 | 0.08 | 581.5 | 0.036 |
|  |  | Weevil-infested | 40 | 22.07 (19.00, 28.01) | 23.68 |  |  |  |  |
|  | QD | Intact | 45 | 42.72 (28.78, 58.48) | 42.26 | 7.03 | 0.16 | 627.5 | 0.017 |
|  |  | Weevil-infested | 40 | 49.75 (41.09, 73.82) | 40.74 |  |  |  |  |
|  | QF | Intact | 20 | 112.64 (102.62, 120.73) | 12.67 | 22.35 | 0.20 | 75 | 0.001 |
|  |  | Weevil-infested | 20 | 134.99 (121.00, 140.83) | 13.52 |  |  |  |  |
|  | QS | Intact | 45 | 120.59 (104.11, 132.91) | 15.44 | 11.59 | 0.10 | 723 | 0.020 |
|  |  | Weevil-infested | 45 | 132.18 (121.14, 143.23) | 15.08 |  |  |  |  |
| Total flavonoids (mg/g) | CD | Intact | 55 | 9.41 (8.24, 14.30) | 45.81 | 3.39 | 0.36 | 915.5 | 0.003 |
|  |  | Weevil-infested | 50 | 12.81 (10.73, 18.08) | 33.22 |  |  |  |  |
|  | CO | Intact | 40 | 13.53 (10.66, 16.64) | 23.32 | 4.14 | 0.31 | 410 | 0.000 |
|  |  | Weevil-infested | 40 | 17.67 (13.74, 19.88) | 30.89 |  |  |  |  |
|  | LD | Intact | 40 | 21.99 (20.28, 23.78) | 11.46 | 3.03 | 0.14 | 502 | 0.004 |
|  |  | Weevil-infested | 40 | 25.03 (21.77, 27.39) | 14.15 |  |  |  |  |
|  | QD | Intact | 45 | 40.55 (34.58, 54.40) | 32.75 | 7.70 | 0.19 | 596.5 | 0.008 |
|  |  | Weevil-infested | 40 | 48.24 (38.90, 73.07) | 33.08 |  |  |  |  |
|  | QF | Intact | 20 | 48.70 (45.64, 56.48) | 17.75 | 22.99 | 0.47 | 54 | 0.000 |
|  |  | Weevil-infested | 20 | 71.69 (59.49, 85.97) | 21.32 |  |  |  |  |
|  | QS | Intact | 45 | 28.33 (23.53, 34.85) | 30.21 | 10.68 | 0.38 | 566 | 0.000 |
|  |  | Weevil-infested | 45 | 39.01 (31.32, 43.81) | 27.31 |  |  |  |  |
| Tannins (mg/g) | CD | Intact | 55 | 1.51 (1.12, 1.75) | 41.58 | 0.82 | 0.54 | 661.5 | 0.000 |
|  |  | Weevil-infested | 50 | 2.32 (1.69, 3.27) | 41.75 |  |  |  |  |
|  | CO | Intact | 40 | 0.83 (0.68, 0.95) | 32.12 | 2.59 | 3.13 | 33 | 0.000 |
|  |  | Weevil-infested | 40 | 3.42 (1.66, 4.10) | 44.69 |  |  |  |  |
|  | LD | Intact | 40 | 4.40 (3.76, 4.91) | 19.87 | 3.53 | 0.80 | 135.5 | 0.000 |
|  |  | Weevil-infested | 40 | 7.93 (6.31, 10.38) | 35.96 |  |  |  |  |
|  | QD | Intact | 45 | 13.22 (11.68, 14.88) | 20.10 | 1.91 | 0.14 | 502 | 0.000 |
|  |  | Weevil-infested | 40 | 15.13 (13.25, 18.82) | 25.55 |  |  |  |  |
|  | QF | Intact | 20 | 14.92 (13.69, 20.56) | 32.87 | 7.83 | 0.52 | 79 | 0.001 |
|  |  | Weevil-infested | 20 | 22.75 (18.82, 27.89) | 28.00 |  |  |  |  |
|  | QS | Intact | 45 | 13.01 (10.98, 16.96) | 24.74 | 2.90 | 0.22 | 640.5 | 0.003 |
|  |  | Weevil-infested | 45 | 15.91 (14.41, 19.54) | 28.83 |  |  |  |  |
| Starch (mg/g) | CD | Intact | 55 | 388.82 (290.94, 461.57) | 28.51 | -46.46 | -0.12 | 1537.5 | 0.299 |
|  |  | Weevil-infested | 50 | 342.37 (251.21, 433.67) | 35.01 |  |  |  |  |
|  | CO | Intact | 40 | 493.13 (379.33, 560.62) | 22.83 | -236.66 | -0.48 | 1263 | 0.000 |
|  |  | Weevil-infested | 40 | 256.47 (211.18, 432.36) | 47.69 |  |  |  |  |
|  | LD | Intact | 40 | 289.78 (197.60, 445.94) | 44.28 | -59.90 | -0.21 | 934.5 | 0.197 |
|  |  | Weevil-infested | 40 | 229.88 (174.95, 355.66) | 50.93 |  |  |  |  |
|  | QD | Intact | 45 | 373.63 (314.61, 446.67) | 23.50 | -18.11 | -0.05 | 1078.5 | 0.117 |
|  |  | Weevil-infested | 40 | 355.51 (280.87, 423.44) | 30.55 |  |  |  |  |
|  | QF | Intact | 20 | 263.77 (239.52, 315.20) | 22.96 | -39.15 | -0.15 | 283 | 0.026 |
|  |  | Weevil-infested | 20 | 224.62 (184.74, 273.85) | 22.18 |  |  |  |  |
|  | QS | Intact | 45 | 325.13 (287.15, 354.35) | 15.08 | 7.60 | 0.02 | 937 | 0.545 |
|  |  | Weevil-infested | 45 | 332.73 (301.17, 367.79) | 18.81 |  |  |  |  |
| Water content (%) | CD | Intact | 229 | 27.07 (18.83, 29.82) | 32.61 | -11.74 | -0.43 | 18163 | 0.000 |
|  |  | Weevil-infested | 101 | 15.33 (10.22, 20.87) | 43.38 |  |  |  |  |
|  | CO | Intact | 83 | 29.50 (25.76, 32.10) | 23.82 | -3.37 | -0.11 | 7311.5 | 0.055 |
|  |  | Weevil-infested | 153 | 26.13 (18.18, 33.33) | 40.45 |  |  |  |  |
|  | LD | Intact | 278 | 27.25 (23.97, 33.13) | 22.59 | -4.68 | -0.17 | 6966.5 | 0.139 |
|  |  | Weevil-infested | 44 | 22.58 (16.14, 38.40) | 48.58 |  |  |  |  |
|  | QD | Intact | 88 | 39.26 (33.62, 42.43) | 27.42 | -26.30 | -0.67 | 13485 | 0.000 |
|  |  | Weevil-infested | 176 | 12.96 (11.32, 25.58) | 57.07 |  |  |  |  |
|  | QF | Intact | 112 | 28.54 (24.96, 31.49) | 29.14 | -17.82 | -0.62 | 6984 | 0.000 |
|  |  | Weevil-infested | 76 | 10.72 (9.55, 17.52) | 54.71 |  |  |  |  |
|  | QS | Intact | 186 | 32.52 (28.22, 34.78) | 21.23 | -21.03 | -0.65 | 24290.5 | 0.000 |
|  |  | Weevil-infested | 158 | 11.49 (10.20, 17.63) | 65.82 |  |  |  |  |

Note: The abbreviation for species is the same as above.

**Table S9.** Results of standardized major axis estimation for the acorn functional traits of different family Fagaceae species.

| Trait(y~x) | Acorn status | R^2^ | *P* | Intercept | Slope | *P*_1.0_ | Common slope | *P*_1.0_ | Shift along common slope *P* |
| --- | --- | --- | --- | --- | --- | --- | --- | --- | --- |
| TP-TF | Intact | 0.62 | <0.001 | -0.705ns | 1.613ns | <0.001 | 1.545(1.377~1.733) | <0.001 | 0.139 |
|  | Weevil-infested | 0.7 | <0.001 | -0.574ns | 1.492ns | <0.001 |  |  |  |
| TP-TA | Intact | 0.83 | <0.001 | 0.957a | 0.802b | <0.001 | - | - | - |
|  | Weevil-infested | 0.79 | <0.001 | 0.757b | 0.944a | 0.39 |  |  |  |
| TF-TA | Intact | 0.81 | <0.001 | 1.031ns | 0.497b | <0.001 | - | - | - |
|  | Weevil-infested | 0.85 | <0.001 | 0.892ns | 0.633a | <0.001 |  |  |  |
| TP-ST | Intact | 0.13 | 0.01 | 10.942ns | -3.719ns | <0.001 | -3.524(-4.257~-2.916) | <0.001 | 0.035 |
|  | Weevil-infested | 0.03 | 0.26 | 10.151ns | -3.438ns | <0.001 |  |  |  |
| TF-ST | Intact | 0.11 | 0.012 | 7.223ns | -2.306ns | <0.001 | -2.281(-2.747~-1.893) | <0.001 | 0.019 |
|  | Weevil-infested | 0.08 | 0.045 | 7.188ns | -2.304ns | <0.001 |  |  |  |
| TA-ST | Intact | 0.15 | 0.005 | 12.449ns | -4.637ns | <0.001 | -4.191(-5.033~-3.489) | <0.001 | 0.006 |
|  | Weevil-infested | 0.08 | 0.047 | 9.948ns | -3.641ns | <0.001 |  |  |  |
| PT-TP | Intact | 0.16 | 0.004 | 0.145ns | -0.285ns | <0.001 | -0.321(-0.353~-0.252) | <0.001 | 0.063 |
|  | Weevil-infested | 0.11 | 0.018 | 0.173ns | -0.315ns | <0.001 |  |  |  |
| PT-TF | Intact | 0.01 | 0.582 | 0.346ns | -0.460ns | <0.001 | -0.467(-0.568~-0.384) | <0.001 | 0.018 |
|  | Weevil-infested | 0.01 | 0.439 | 0.353ns | -0.470ns | <0.001 |  |  |  |
| PT-TA | Intact | 0.04 | 0.174 | -0.128ns | -0.229ns | <0.001 | -0.254(-0.309~-0.209) | <0.001 | 0.005 |
|  | Weevil-infested | 0 | 0.851 | -0.065ns | -0.297ns | <0.001 |  |  |  |
| CIT-TP | Intact | 0.13 | 0.008 | -0.465b | 0.264ns | <0.001 | 0.260(0.217~0.312) | <0.001 | 0.945 |
|  | Weevil-infested | 0.22 | <0.001 | -0.500a | 0.256ns | <0.001 |  |  |  |
| CIT-TF | Intact | 0.16 | 0.004 | -0.652a | 0.426ns | <0.001 | 0.402(0.336~0.483) | <0.001 | 0.741 |
|  | Weevil-infested | 0.19 | 0.001 | -0.647b | 0.383ns | <0.001 |  |  |  |
| CIT-TA | Intact | 0.16 | 0.003 | -0.212b | 0.212ns | <0.001 | 0.219(0.183~0.262) | <0.001 | 0.45 |
|  | Weevil-infested | 0.31 | <0.001 | -0.306a | 0.242ns | <0.001 |  |  |  |
| PT-ST | Intact | 0 | 0.687 | 2.432a | -1.061ns | 0.681 | -1.064(-1.295~-0.875) | 0.782 | 0.836 |
|  | Weevil-infested | 0.07 | 0.06 | 2.389b | -1.082ns | 0.57 |  |  |  |
| CIT-ST | Intact | 0 | 0.712 | 2.427a | -0.983ns | 0.906 | -0.918(-1.118~-0.754) | 0.676 | 0.446 |
|  | Weevil-infested | 0.01 | 0.53 | 2.104b | -0.882ns | 0.38 |  |  |  |

Note: In intercept and slope column, different letters between sound acorns and infested acorns indicate there are significant differences between them (*P* < 0.05). P1.0 indicates the significant difference between absolute value of the common slope and 1, and *P* < 0.05 shows evident difference and there are allometric growth relationship between functional traits.
